# Genetic relationships between the RACK1 homolog *cpc-2* and heterotrimeric G protein subunit genes in *Neurospora crassa*

**DOI:** 10.1371/journal.pone.0223334

**Published:** 2019-10-03

**Authors:** Amruta Garud, Alexander J. Carrillo, Logan A. Collier, Arit Ghosh, James D. Kim, Berenise Lopez-Lopez, Shouqiang Ouyang, Katherine A. Borkovich

**Affiliations:** Department of Microbiology and Plant Pathology, University of California, Riverside, California, United States of America; Oregon State University, UNITED STATES

## Abstract

Receptor for Activated C Kinase-1 (RACK1) is a multifunctional eukaryotic scaffolding protein with a seven WD repeat structure. Among their many cellular roles, RACK1 homologs have been shown to serve as alternative Gβ subunits during heterotrimeric G protein signaling in many systems. We investigated genetic interactions between the RACK1 homolog *cpc-2*, the previously characterized Gβ subunit *gnb-1* and other G protein signaling components in the multicellular filamentous fungus *Neurospora crassa*. Results from cell fractionation studies and from fluorescent microscopy of a strain expressing a CPC-2-GFP fusion protein revealed that CPC-2 is a cytoplasmic protein. Genetic epistasis experiments between *cpc-2*, the three Gα genes (*gna-1*, *gna-2* and *gna-3*) and *gnb-1* demonstrated that *cpc-2* is epistatic to *gna-2* with regards to basal hyphae growth rate and aerial hyphae height, while deletion of *cpc-2* mitigates the increased macroconidiation on solid medium observed in Δ*gnb-1* mutants. Δ*cpc-2* mutants inappropriately produce conidiophores during growth in submerged culture and mutational activation of *gna-3* alleviates this defect. Δ*cpc-2* mutants are female-sterile and fertility could not be restored by mutational activation of any of the three Gα genes. With the exception of macroconidiation on solid medium, double mutants lacking *cpc-2* and *gnb-1* exhibited more severe defects for all phenotypic traits, supporting a largely synergistic relationship between GNB-1 and CPC-2 in *N*. *crassa*.

## Introduction

Heterotrimeric G protein signaling cascades consist of seven-helix transmembrane G Protein Coupled Receptors (GPCRs) and the three G protein subunits—Gα, Gβ and Gγ [1–3]. In the inactive state, the Gαβγ heterotrimer is associated with the GPCR. Ligand stimulation causes exchange of GDP for GTP on the Gα, leading to dissociation of Gα-GTP from the Gβγ heterodimer. The Gα-GTP and the Gβγ dimer can then regulate downstream effectors, leading to changes in cellular physiology [3]. The Gα-GTP has native GTPase activity that causes release of the inorganic phosphate from the GTP. The Gα-GDP then reassociates with the Gβ subunit and GPCR, leading to signal termination and completion of the cycle.

*Neurospora crassa* is a multicellular ascomycete fungus that has emerged as a model system to study G protein signaling, and comparisons with *N*. *crassa* have driven discoveries in pathogenic fungi and higher eukaryotes [4, 5]. In *N*. *crassa*, there are 43 predicted GPCRs, three Gα subunits (GNA-1, GNA-2 and GNA-3), one characterized Gβ subunit (GNB-1) and one Gγ subunit (GNG-1) [6, 7]. Major processes such as hyphal growth, macroconidiation, conidial germination, mating, nutrient sensing and temperature and oxidative stress resistance are regulated by G protein signaling pathways in *N*. *crassa* [8–14].

Receptor for Activated C Kinase-1 (RACK1) is a major scaffolding protein in many eukaryotic systems. Similar to G protein β subunits, RACK1 has a seven WD repeat structure, and is one of the best-studied proteins in the WD-repeat family [15]. Initially identified as a protein that binds to the active conformation of protein kinase C (PKC) βII, RACK1 is now known to be multifunctional [16, 17]. For example, RACK1 allows cross talk between the PKC and Mitogen Activated Kinase (MAP) pathways by acting as a scaffold for the Jun N-terminal Kinase (JNK) upon stimulation, leading to PKC-mediated phosphorylation and activation of JNK [18]. It has been observed that RACK1 binds to the Gβγ dimer in HEK293 cells and also regulates a subset of its functions, including promoting its dislocation from the cytosol to the membrane [19]. Additionally, RACK1 is known to associate with the 40S subunit of the ribosome, near the mRNA exit channel [20]. Due to its conformation when bound to the ribosome, RACK1 is believed to serve as an adaptor, bringing together proteins at the ribosome during translation [reviewed in [15]].

Homologs of RACK1 have been implicated as alternative Gβ subunits in the fungal kingdom, through direct interaction with Gα subunits [21, 22]. In *Saccharomyces cerevisiae*, Asc1p functions as a Guanine nucleotide Dissociation Inhibitor (GDI) for the Gα Gpa2, and is involved in regulating glucose responsiveness through its binding to adenylyl cyclase (Cyr1) [23]. *gib2*, an essential gene in *Crytpococcus neoformans*, encodes a protein that binds to the Gα Gpa1 and two Gγ subunits, Gpg1 and Gpg2. It also associates with Smg1, a downstream target of cAMP signaling, and to the protein kinase C homolog Pkc1 [24]. In *Magnaporthe oryzae*, the RACK1 ortholog MoMip11 interacts with the Gα protein MoMagA and the Regulator of G protein Signaling (RGS) protein MoRgs7 to regulate pathogenicity [25, 26].

Additional RACK1 orthologs have been shown to regulate various aspects of growth and development in several fungal systems, but without demonstration of a physical interaction with heterotrimeric Gα proteins. *S*. *pombe* Cpc2 plays a role in cell cycle regulation and stress responses through ribosomal association [27] and translational control of the stress response transcriptional factor Atf1 [27]. RACK1 orthologs from *Aspergillus nidulans* and *Aspergillus fumigatus* have been demonstrated to regulate sexual differentiation and asexual growth and development, respectively [28, 29]. In *Ustilago maydis*, Rak1 is essential for the transcription of *rop*1, which is a direct positive regulator of the pheromone response factor (*prf1*), making it essential for mating [30]. Strains lacking RAK1 also have attenuated filamentation and virulence, and abnormal cell morphology [30].

The *N*. *crassa* RACK1 homolog CPC-2 was the first reported RACK1 protein in fungi, initially identified as a component of the general amino acid regulation network [31]. In *N*. *crassa*, starvation for a single amino acid leads to an overall derepression of all amino acid biosynthetic genes at the level of transcription [32]. Loss of the *cpc-2* gene blocks derepression of amino acid biosynthetic genes during amino acid limiting conditions [31]. Under non-starved conditions, loss of the *cpc-2* gene decreases growth by 50% [33]. During the sexual cycle, the Δ*cpc-2* mutant lacks protoperithecia, and is female-sterile [33]. Other components of this cross pathway control network are *cpc-1*, homologous to *GCN4* [34], and *cpc-3*, the *N*. *crassa* equivalent of *GCN2* [35]. Analysis of Δ*cpc-2* Δ*cpc-3* and Δ*cpc-2* Δ*cpc-1* double mutants showed that they possessed Δ*cpc-2* phenotypes, such as reduced growth and female sterility. These findings suggested that *cpc-2* has broader functions operating outside of amino acid control [35].

To-date, no one has explored a possible function for CPC-2 in G protein signaling in *N*. *crassa*. In this study, we use strains carrying single and double gene deletions or expressing constitutively activated Gα alleles to analyze genetic epistasis between components of the G protein pathway and the *cpc-2* gene. We produce a polyclonal antibody against CPC-2 and use western analysis to determine protein levels in the mutants lacking the other G protein subunits. Our results reveal that *N*. *crassa* mutants lacking both predicted Gβ subunits are viable, but possess major defects in growth and development. We also provide evidence for G protein dependent and independent functions for CPC-2 in *N*. *crassa*.

## Materials and methods

### Strains and media

*N*. *crassa* strains were either obtained from the Fungal Genetics Stock Center (FGSC; Kansas State University, Manhattan, KS) [36] or created during this work (Table 1). Strains that are not deposited in the FGSC collection are available upon request. Strains were cultured in Vogel’s minimal medium (VM) [37] to propagate vegetative hyphae or asexual spores (macroconidia; conidia). Synthetic Crossing Medium (SCM) plates containing 1% agar were used to induce development of female sexual reproductive structures [38]. Sorbose-containing medium (FGS) was used to facilitate colony formation on plates [39]. Media was supplemented with 100 μg/ml of histidine, 10 μg/ml pantothenate, 200 μg/ml hygromycin (Calbiochem, San Diego, CA), 200 μg/ml nourseothricin (Werner BioAgents, Germany) or 400 μg/ml phosphinothricin (purified from Finale, Farnam Companies, Inc., Phoenix, AZ), where indicated. Conidia were propagated in VM agar flasks as described previously [39]. Liquid cultures were brought to a concentration of 1x10^6^ conidia/ml and incubated with shaking at 200 RPM at 30°C for 16 hr. *Escherichia coli* strain DH5α was used to maintain all plasmids.

**Table 1:** *N*. *crassa* strains used in this study.

Strain name	Relevant genotype	Comments	Source or Reference
74-OR23-1VA	Wild type, *mat A*		FGSC^a^ 2489
ORS-SL6a	Wild type, *mat a*		FGSC4200
74A-OR23-1A	Wild type, *mat A*		FGSC987
a^m1^	*cyh-1*, *ad3B*, *a*^*m1*^		FGSC4564
Y234M723	*his-3*, *mat A*		FGSC6103
his-3a#14	*his-3*, *mat a*		Ref. [61]
3B10	Δ*gna-1*::*hph*^+^, *mat a*		Ref. [81]
Δgna2-2477	Δ*gna-2*::*hph*^+^, *mat a*		FGSC12377
Δgna2-2476	Δ*gna-2*::*hph*^+^, *mat A*		FGSC12376
31c2	Δ*gna-3*::*hph*^+^, *mat A*		Ref. [14]
42-8-3	Δ*gnb-1*::*hph*^+^, *mat A*		Ref. [64]
Δcpc2Het	Δ*cpc-2*::*hph*^+^, Δ*mus-51*::*bar*^+^, *mat a* (heterokaryon)		FGSC13695
Δcpc2#1	Δ*cpc-2*::*hph*^+^, Δ*mus-51*::*bar*^+^, *mat a*	Progeny from cross of Δcpc2Het to 74-OR23-1VA	This Study
Δcpc2#6	Δ*cpc-2*::*hph*^+^, Δ*mus-51*::*bar*^+^, *mat a*	Progeny from cross of Δcpc2Het to 74-OR23-1VA	This Study
Δcpc2#11	Δ*cpc-2*::*hph*^+^, *mat A*	Progeny from cross of Δcpc2Het to 74-OR23-1VA	This Study
Δcpc2his3A	Δ*cpc-2*::*hph*^+^, *his-3*, *mat A*	Progeny from cross of Δcpc2Het to Y234M723	This Study
cpc2+*a*^*m 1*^	Δ*cpc-2*::*hph*^+^, *his-3*, *mat A + a*^*m 1*^, *cyh-1*, *ad3B*, *mat A* (heterokaryon)	Heterokaryon of Δcpc2his3A and a^m1^	This Study
C2G1*#44	Δ*cpc-2*::*hph*^+^, *his-3*^+^::*gna-1*^Q204L^, *mat A*	Δcpc2his3A purified transformant	This Study
C2G2*#4	Δ*cpc-2*::*hph*^+^, *his-3*^+^::*gna-2*^Q205L^, *mat A*	Δcpc2his3A purified transformant	This Study
C2G3*#1–8	Δ*cpc-2*::*hph*^+^, *his-3*^+^::*gna-3*^Q208L^, *mat A*	Δcpc2his3A purified transformant	This Study
C2G1#39	Δ*cpc-2*::*hph*^+^, Δ*gna-1*::*hph*^+^, *mat a*	Progeny from cross of cpc2+*a*^*m 1*^ to 3b10	This Study
C2G2#37	Δ*cpc-2*::*hph*^+^, Δ*gna-2*::*hph*^+^, *mat a*	Progeny from cross of cpc2+*a*^*m 1*^ to Δgna2-2477	This Study
C2G3#1–6	Δ*gna-3*::*hph*^+^, Δ*cpc-2*::*hph*^+^, *mat A*	Progeny from cross of 31c2 to Δcpc2#6	This Study
C2B1#2-1-1	Δ*gnb-1*::*nat*^+^, Δ*cpc-2*::*hph*^+^, *mus-51*::*bar*^+^, *mat a*	Δcpc2#6 purified transformant	This Study
51–4	Δ*rid*::*nat*^+^, Δ*mus-51*::*nat*^+^, *mat a*		This Study
CPC-2-GFP-9	Δ*pan-2*::p*ccg-1*::*cpc-2*-V5-GFP::*bar*^+^, *mat a*	51-4-1 transformant	This Study
CPC-2-GFP-9-10	Δ*pan-2*::p*ccg-1*::*cpc-2*-V5-GFP::*bar*^+^, *mat a*	Progeny of CPC-2-GFP-9 crossed to 74-OR23-1VA	This Study
pccg-1_GFP	Δ*pan-2*::p*ccg-1*::V5-GFP::*bar*^+^, *mat a*	Empty vector control for CPC-2-GFP-9-10	Ref. [58]
CPC-2-GFP-13	Δ*pan-2*::p*ccg-1*::*cpc-2*-V5-GFP::*bar*^+^, *mat a*	51-4-1 transformant	This Study
CPC-2-GFP-13.2	Δ*cpc-2*::*hph*^+^, Δ*pan-2*::p*ccg-1*::*cpc-2*-V5-GFP::*bar*^+^, *mat a*	Progeny of CPC-2-GFP-13 crossed to 74-OR23-1VA	This Study

^a^FGSC: Fungal Genetics Stock Center, Kansas State University, Manhattan, KS [36]

### Phylogenetic analysis

**Fig 1:**
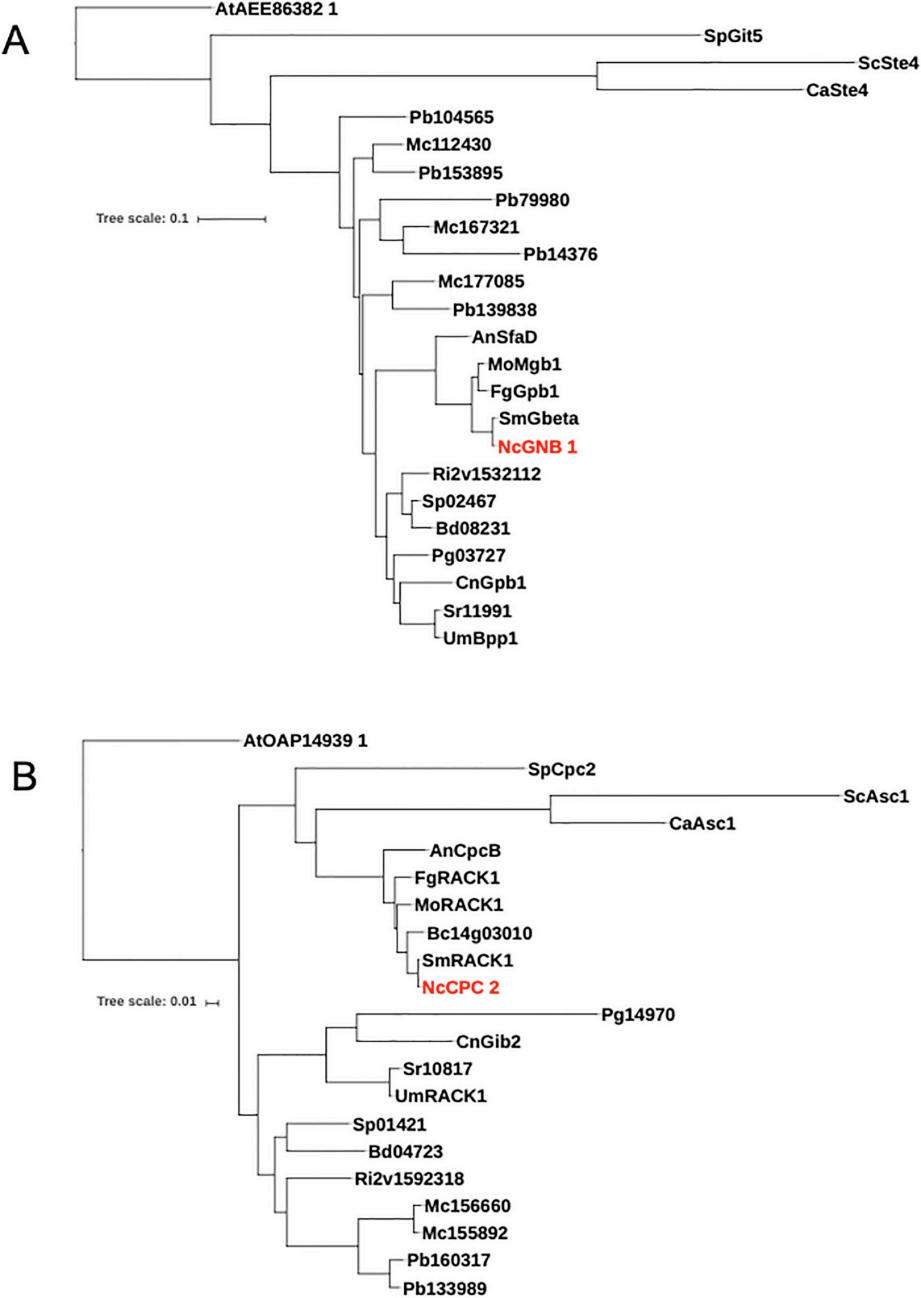
Phylogenetic analysis of Gβ and RACK1 proteins from 10 fungal species. Amino acid sequences were obtained from FungiDB or NCBI and phylogenetic analysis conducted using the “One-Click Workflow” tool at NGPhylogeny.fr. The final trees were drawn using tools at itol.embl.de (see Materials and methods for details). **A. Gβ proteins**. Organisms and protein names/accession numbers for the Gβ orthologs are *Neurospora crassa* NcGNB-1/NCU00440; *Sordaria macrospora* SmGbeta/SMAC01876; *Fusarium graminearum* GzGPB1/FGRAMPH101G14499; *Magnaporthe oryzae* MoMgb1/MGG05201; *Aspergillus nidulans* AnSfaD/AN0081; *Ustilago maydis* UmBpp1/UMAG00703; *Cryptococcus neoformans* CnGpb1/CNAG01262; *Candida albicans* CaSte4/C204210WA; *Schizosaccharomyces pombe* SpGit5/SPBC32H8.07 and *Saccharomyces cerevisiae* ScSte4/YOR212W; *Batrachochytrium dendrobatidis*/BDEG_08231; *Botrytis cinere*a/Bcin08g01420; *Puccinia graminis f*. *sp*. *tritici*/PGTG_03727; *Sporisorium reilianum*/sr11991; *Spizellomyces punctatus*/SPPG_02467; *Phycomyces blakesleeanus*/PHYBL_104565; *Phycomyces blakesleeanus*/PHYBL_139838; *Phycomyces blakesleeanus*/PHYBL_14376; *Phycomyces blakesleeanus*/PHYBL_153895; *Phycomyces blakesleeanus*/PHYBL_79980; *Mucor circinelloides f*. *lusitanicus*/QYA_112430; *Mucor circinelloides f*. *lusitanicus/*QYA_167321; *Mucor circinelloides f*. *lusitanicus*/QYA_177085; *Rhizophagus irregularis/*GLOIN_2v1532112; *Arabidopsis thaliana*/AEE86382.1 **B. RACK1 proteins**. Organisms and protein names or accession numbers for the RACK1 orthologs are *Neurospora crassa* NcCPC-2/NCU05810; *Sordaria macrospora* SmRACK1/SMAC07639; *Fusarium graminearum* FgRACK1/FGRAMPH101G06721; *Magnaporthe oryzae* MoRACK1/MGG04719; *Aspergillus nidulans* AnCpcB/AN4163; *Ustilago maydis* UmRACK1/UMAG10146; *Cryptococcus neoformans* CnGib2/CNAG05465; *Candida albicans* CaAsc1/C701250WA; *Schizosaccharomyces pombe* SpCpc2/SPAC6B12.15; *Saccharomyces cerevisiae* ScAsc1/YMR116C; *Batrachochytrium dendrobatidis*/BDEG_04723; *Botrytis cinerea*/Bcin14g03010; *Sporisorium reilianum*/sr10817; *Puccinia graminis f*. *sp*. *tritici*/PGTG_14970; *Phycomyces blakesleeanus*/PHYBL_133989; *Phycomyces blakesleeanus*/PHYBL_160317; *Mucor circinelloides f*. *lusitanicus*/QYA_155892; *Mucor circinelloides f*. *lusitanicus*/QYA_156660; *Rhizophagus irregularis*/GLOIN_2v1592318; *Arabidopsis thaliana*/AT OAP14939.1.

Protein sequences orthologous to *N*. *crassa* CPC-2 (NCU05810) and GNB-1 (NCU00440) from 18 fungal species chosen to represent a diversity of fungi [40] were obtained from the FungiDB database (fungidb.org) [41]. Sequences for the Gβ and RACK1 proteins from the plant *Arabidopsis thaliana* were retrieved from the National Center for Biotechnology Information (NCBI). The “One-Click Workflow” tool at NGPhylogeny.fr [42] was implemented for the phylogenetic analysis. This pipeline uses FASTA files to generate a multiple alignment using MAFFT (**M**ultiple **A**lignment using **F**ast **F**ourier Transform) [43]. Alignments were inspected and proteins from the 18 species resulted in good alignments for both the Gβ and RACK1. The MAFFT alignments were curated using BMGE (**B**lock **M**apping and **G**athering with **E**ntropy) [44] and FastME (Fast **M**inimum **E**volution) [45] was used to produce the tree file. FastME uses distance algorithms to infer phylogenies. The final trees were drawn using tools at the Interactive Tree of Life (iTOL; itol.embl.de) [46]. Species and gene accession numbers are in the legend to Fig 1.

### *N*. *crassa* strain construction

The Δ*cpc-2*::*hph*^*R*^ knockout mutant was deposited at the FGSC as a heterokaryon (FGSC13695). Homokaryotic mutants were obtained from the heterokaryon after a sexual cross to wild type strain 74-OR23-1VA and plating ascospores on medium containing hygromycin. Progeny were checked using diagnostic PCR [47] with *cpc-2* (Primer 1 or Primer 2) and *hph* (Primer 13 or Primer 14) primers (Table 2; S1 Fig), and then spot-tested on phosphinothricin to check for the presence of the *mus-51* mutation, which is marked with *bar* [48, 49].

**Table 2:** Oligonucleotides used in this study.

Primer #	Primer name	Sequence 5’ to 3’
1	CPC2FORDIAG	AGCAGGGCCGGGTGGAGATT
2	CPC2REVDIAG	CGAAGGTCCCACCCTAACAGCC
3	GNA1FORDIAG-1	CTTGGAGAGTGCGCGGTGGG
4	GNA1FORDIAG-2	GTCGGGTGGGCGATGGATCAA
5	GNA1REVDIAG	GTGTCGGGTGCTTTCTGCCA
6	GNA2FORDIAG-1	GCCCTGGGCACTACCGAAACG
7	GNA2FORDIAG-2	GGGCCAGAAATGGAACCTACC
8	GNA2REVDIAG	TTCCGGCCGAGTGAAACGCT
9	GNA3FORDIAG	GCGGCCTGCCCTAGCAATTCA
10	GNA3REVDIAG	GGAGTAGCGAGGTGTATGAGTGGT
11	GNB1FORDIAG	GTGCCTTCGGCCAGGCTTGT
12	GNB1REVDIAG	TTGGTTACGTATGCTGAGCAAGGG
13	HPHREV	TGCTCCTTCAATATCATCTTCTGTC
14	HPHFOR	TGTGTAGAAGTACTCGCCGATAGTG
15	GNB1NAT5’FLANK-FWD	GTAACGCCAGGGTTTTCCCAGTCACGACGGTTCCATCGGGGGTGCGGTGC
16	GNB1NAT5’FLANK-REV	CTACATGAGCATGCCCTGCCCCTGATCGCTTCTGCGAGTGGGCGGGCGGC
17	GNB1NAT3’FLANK-FWD	CTCCTTCAATATCATCTTCTGTCGAGCTCTCGCTTGTATGCATCAGGTCT
18	GNB1NAT3’FLANK-REV	GCGGATAACAATTTCACACAGGAAACAGCAAGGGCACTGGCCCACTGGAC
19	PTRPC5’GNB1	AGACCTGATGCATACAAGCGAGAGCTCGACAGAAGATGATATTGAAGGAG
20	NAT3’GNB1	GCCGCCCGCCCACTCGCAGAAGCGATCAGGGGCAGGGCATGCTCATGTAG
21	GNB1NAT-REV-DIAG	TCACGTAGACCTGATGCATA
22	NAT 5’ REV DIAG	CAAAAAGTGCTCCTTCAATA
23	pCCG1 FWD	CCATCATCAGCCAACAAAGC
24	GNA1ORF REV	GGGAATTCTCAAATCAAACCGCAG
25	GNA2ORF REV	GGGAATTCCTACAGGATAAGTTGT
26	GNA3ORF REV	GGGAATTCTCATAGAATACCGGAG
27	F-C2	CCACTTTCACAACCCCTCACATCAACCAAAATGGCTGAGCAACTCATCCTCAAG
28	R-C2-V5G	GTTAGGGATAGGCTTTCCGCCGCCTCCGCCAGCGCGGGACATGACACCCCAGGC
29	P-CPC-2-REV	TATGCTAGTTATGCGGCCGCTGCAGTTAAGCGCGGGACATGACACCCCAG

Double mutants Δ*cpc-2*, Δ*gna-1*; Δ*cpc-2*, Δ*gna-2* and Δ*cpc-2*, Δ*gna-3* were made using genetic crosses between single mutants [39] (Table 1). In cases where both single mutants in the cross were female-sterile (Δ*cpc-2* and Δ*gna-1*), the strain used as the female was a heterokaryon with the *a*^m1^ helper strain [50]. The presence of the mutations in the progeny was verified by diagnostic PCR using pairs of gene-specific and *hph* cassette-specific primers (Primers 1–14 in Table 2; S1 Fig).

Repeated attempts to generate a Δ*cpc-2* Δ*gnb-1* double mutant through a sexual cross were unsuccessful. Therefore, the double mutant was created by electroporation of the Δcpc2#6 strain using a knockout cassette for *gnb-1*, marked with nourseothricin resistance (*nat*^R^) [51]. The Δ*gnb-1* knockout cassette was created using yeast recombinational cloning in vector pRS426 [52], with methods previously described [53]. Primers used to amplify fragments for the construct are listed in Table 2. Primer pairs 15–16 and 17–18 were used to amplify the 1 kb 5’ and 3’ flanks of *gnb-1*, respectively, from genomic DNA. Primers 19 and 20 were used to amplify the nourseothricin resistance marker from plasmid pD-NAT-1 [51]. The three purified PCR products plus pRS426 digested with *Xho*I and *Eco*RI were transformed into yeast strain FY834 [54]. Transformants were selected on FGS plates containing nourseothricin and then checked for the presence of the Δ*gnb-1*::*nat*^R^ mutation using diagnostic PCR with gene-specific primers (Primers 21 and 22; Table 2). Positive strains were then purified to homokaryons using serial streaking of macroconidia [47] and checked again using diagnostic PCR (S1 Fig).

Vectors containing predicted GTPase-deficient, constitutively activating mutations *gna-1*^Q204L^ (pSVK51), *gna-2*^Q205L^ (pSVK52), and *gna-3*^Q208L^ (pSVK53) were previously made using site-directed mutagenesis [55]. Electroporation of *N*. *crassa* with 1–2 μg of pSVK51, pSVK52 or pSVK53 was as previously described [56], using the Δcpc2his3A strain as the recipient, with selection on FGS plates without histidine. Genomic DNA was extracted from transformants and subjected to Southern analysis for *gna-1*, *gna-2* and *gna-3* as described [55]. Transformants determined to have a single integration event of the transforming DNA at the *his-3* locus were purified to homokaryons using microconidiation [57] or serial streaking of macroconidia [47] on FGS plates lacking histidine. Genomic DNA was extracted from these strains and analyzed using diagnostic PCR (S1 Fig) to confirm genotypes.

A vector was produced to allow expression of a GFP-tagged version of *cpc-2* in *trans* to the wild-type copy. The vector backbone (pRS426PVG) [58] was assembled in plasmid pRS426 using yeast recombinational cloning [53]. The fragments included a region 1kb 5’ of the *pan-2* ORF, the *ccg-1* promoter amplified from pMF272 [59], a multiple cloning sequence, a 5xGlycine linker, a V5-tag, GFP sequence amplified from pMF272 [59], the *bar* gene, amplified from vector pTJK1 [60] and a 1 kb fragment 3’ of the *pan-2* ORF [58]. All fragments were amplified using Phusion High-Fidelity DNA Polymerase (New England Biolabs, Ipswich, MA). The *pan-2* flanking sequences allow targeting to, and deletion of, the *pan-2* ORF, resulting in pantothenate auxotrophy. The final expression construct for CPC-2 was produced by insertion of the *cpc-2* ORF (amplified using Primers 27 and 28; Table 2) into vector pRS426PVG (linearized using *Pac*I) using yeast recombinational cloning [53]. Vector pRS426PVG-CPC2 was transformed using electroporation into *N*. *crassa* strain 51-IV-4 (Table 1) [58]. Transformants were selected on medium containing phosphinothricin and pantothenate [58] and screened for the presence of the inserted DNA at the *pan-2* locus using PCR. Positive strains were crossed to wild-type strain 74-OR23-1VA, and ascospores were plated on medium containing phosphinothricin and pantothenate. Progeny were screened for panthothenate auxotrophy by spot-testing and for the presence of the integrated DNA using diagnostic PCR (S1 Fig). Strain CPC-2-GFP-9-10 was selected for further study (Table 1).

A *cpc-2* complemented strain was obtained by crossing the transformants expressing GFP-tagged *cpc-2* described above to Δ*cpc-2* mutant strain Δcpc2#11 (Table 1). Ascospores were plated on FGS plates containing hygromycin and pantothenate to select strains carrying the Δ*cpc-2* mutation. Progeny were spot-tested on medium containing phosphinothricin and panthothenate, followed by diagnostic PCR (S1 Fig), to determine those that also carried the *cpc-2* GFP trans gene construct at the *pan-2* locus. Positive strains were tested for the presence of the CPC-2 GFP fusion protein using western analysis with CPC-2 antiserum as described below. Strain CPC-2-GFP-13.2 was selected for further analysis (Table 1).

### Purification of a CPC-2 fusion protein for production of a polyclonal antiserum in rabbits

CPC-2 was expressed as an in-frame, N-terminal Maltose Binding Protein (MBP) fusion protein in *E*. *coli* and then purified and used as an antigen for antibody generation in rabbits. The *cpc-2* ORF was cloned as an *EcoR*I-*Pst*I fragment in *E*. *coli* vector pMAL-c2X (New England Biolabs). The MBP-CPC-2 fusion protein was expressed in *E*. *coli* strain K12 ER2508 (New England Biolabs) with induction using 300 μM IPTG (isopropyl β-D-1-thiogalactopyranoside; Sigma) and the fusion protein purified using an amylose resin according to the manufacturer’s recommendations. A polyclonal antiserum specific for the MBP-CPC-2 protein was raised in rabbits by Cocalico Biologicals, Inc. (Stevens, PA, USA).

### Western analysis to confirm genotypes and check protein levels in mutants

Western analysis was used to check strains for expression of CPC-2 and the G protein subunits GNA-1, GNA-2, GNA-3 and GNB-1. For confirming genotypes, submerged cultures were grown, frozen in liquid nitrogen and then pulverized in 2-ml tubes with metal beads using a TissueLyser (Qiagen Retsch GbmH, Hannover, Germany) as previously described [47]. Subsequently, 500–800 μl of extraction buffer (10mM TrisCl pH 7.5, 0.5 mM EDTA, 0.1% Fungal Protease Inhibitor Cocktail (FPIC), 1 mM PMSF and 1mM DTT) was added to the tube, the solution was mixed and then centrifuged at 5000 x g for 10 min at 4°C. Protein concentration was determined using the Bradford Protein Reagent Concentrate (Bio-Rad, Hercules, CA). Approximately 50 μg of supernatant protein (whole cell extract) was loaded onto a 10% SDS-PAGE gel and then transferred to a nitrocellulose membrane (GE Water and Process Technologies) [61]. For checking G protein levels in the Δ*cpc-2* mutant, cultures were grown and the protein fraction enriched in plasma membranes was isolated as previously described [62]. For determining CPC-2 protein amount in the G protein mutants, whole cell extracts were isolated as previously described [62]. The protein concentration in the preparations was determined using the Bradford Protein Reagent Concentrate. Aliquots containing equal amounts of protein were subjected to SDS-PAGE, and a western blot was prepared as described above for confirming genotypes of G protein subunit mutants.

Western blot membranes were reacted with the CPC-2 antibody at a dilution of 1:1000 or antiserum raised against GNA-1, GNA-2, GNA-3 or GNB-1 at dilutions of 1:2000 [55, 56, 63, 64]. Blots were then incubated with a goat anti-rabbit antibody horseradish peroxidase conjugate (Bio-Rad; 1:10,000 dilution). Chemiluminescent detection was performed as previously described [61] using the Super Signal West Pico Plus kit (Thermo Fisher, Rockford, IL). Western blots presented in figures are representative of three biological replicates.

### Phenotypic analysis

Quantitative assays for aerial hyphae height and growth rates of basal hyphae and qualitative analysis of female fertility were performed as described previously [6, 65]. Twelve biological replicates were obtained for aerial hyphae height and four were used for basal hyphae growth rate calculations. Investigation of hyphal morphology and conidiation in submerged cultures and conidial germination on solid medium were conducted as described previously [55, 66] and the results shown are representative of 2–3 biological replicates. Because the Δ*cpc-2 gna-3*^Q208L^ strain KAB3210 does not produce appreciable macroconidia, 200 microliters of packed aerial hyphae were used to inoculate submerged cultures for this strain. For quantifying macroconidia, strains were inoculated in 13x100mm glass slant tubes containing 3 ml of VM agar medium and incubated for 4 days in the dark at 30°C and 3 days in light at room temperature. Macroconidia were collected from tubes by adding 2 ml water, mixing vigorously using a vortex mixer and filtering through Handiwipes into a 15 ml conical tube using a small funnel. This step was repeated twice, once using a wooden stick to dislodge residual macroconidia from the glass tube prior to vortexing and filtering. Macroconidia were pelleted by centrifugation and the water aspirated. Water was added to an appropriate volume and the absorbance read at 600nm using a spectrophotometer. The readings for different strains were all normalized to the same volume (1 ml) to yield a macroconidial concentration expressed as OD600/ml. Eight biological replicates were obtained.

GraphPad Prism 6.0 (GraphPad Software Inc., La Jolla, CA) was used to analyze quantitative traits (hyphal growth rate, aerial hyphae height and conidia abundance). Grubb’s Q test was utilized to detect and eliminate outliers and then the Ordinary One-Way ANOVA test was used for detecting statistical significance. The p-value cutoff was set to 0.05, confidence intervals were 95% and pair-wise comparisons were made. Graphs were created using Microsoft Excel (Microsoft, Redmond, WA).

### CPC-2 localization experiments

Two approaches were undertaken to determine the intracellular localization of the CPC-2 protein: cell fractionation studies using centrifugation with a wild-type strain and live-cell microscopic imaging of a strain that produces GFP-tagged CPC-2. Cell fractionation of a whole cell extract of strain 74-OR23-IVA (Table 1) was performed as described [55]. Fractions containing whole cell extract, cytosol, and the particulate fraction (membranous organelles and large macromolecular structures) were isolated. The volumes of the cytosol and particulate fractions were adjusted to the same total volume as the original whole cell extract to allow comparison. The protein concentration of the whole cell extract was determined using the Bradford Protein Reagent Concentrate (Bio-Rad). Aliquots containing a volume identical to that containing 50 μg of protein from the whole cell extract were subjected to SDS-PAGE and gels were blotted onto nitrocellulose membranes. Antibody to arginase/AGA (cytosolic marker) [67] was used at a dilution of 1:10,000 and the plasma membrane ATPase/PMA-1 (plasma membrane marker; gift from Kenneth Allen and Clifford Slayman) [68] was used at a dilution of 1:3000. Westerns shown in figures are representative of four biological replicates.

Fluorescence microscopy of the CPC-2-GFP-9-10 strain was conducted essentially as described [66]. The germinating conidia were visualized using differential interference microscopy on an Olympus IX71 inverted microscope (Olympus America) with a 60X oil immersion objective. For visualization of GFP fluorescence, the GFP laser was used for excitation at 400 nm. Images were captured using a QIClickTM digital CCD camera (QImaging Surrey, British Columbia, Canada).

## Results

### *N*. *crassa* CPC-2 is homologous to predicted RACK1 proteins from other fungi

*N*. *crassa* CPC-2 is 316 amino acids in length and was previously reported to have 70% percent identity with RACK1 proteins [33]. *N*. *crassa* CPC-2 and GNB-1 each possess seven WD-40 repeats and share 39% similarity and 24% identity at the protein level. In order to investigate the relationships between CPC-2, GNB-1 and RACK1 and Gβ subunit proteins from other fungi, we subjected orthologous sequences from 18 fungal species to multiple sequence alignment and tree rendering (See Materials and methods for details). Gβ and RACK1 orthologs from the plant *Arabidopsis thaliana* were included as outgroups for the analysis. The fungal species include representatives from the Ascomycota (nine species), Basidiomycota (four species), Chytridiomycota (two species) and Mucoromycota (three species) [40]. Two of the species from the Mucoromycotina possessed multiple orthologs of both GNB-1 and CPC-2, and all proteins were included in our analysis.

The results for the Gβ group showed that the proteins from *N*. *crassa* and the other Ascomycete filamentous fungi (*Sordaria macrospora*, *Fusarium graminearum*, *Botrytis cinerea*, *Magnaporthe oryzae* and *Aspergillus nidulans*) cluster together and are more closely related to proteins from Basidiomycetes (*Ustilago maydis*, *Cryptococcus neoformans*, *Sporisorium reilianum* and *Puccinia graminis f*. *sp*. *tritici*), Chytridiomycetes (*Batrachochytrium dendrobatidis* and *Spizellomyces punctatus*) and Mucoromycetes (*Phycomyces blakesleeanus* and *Mucor circinelloides f*. *lusitanicus*) than to the three Ascomycete yeasts (*Saccharomyces cerevisiae*, *Candida albicans* and *Schizosaccharomyces pombe*) (Fig 1A). These relationships are in keeping with our previous observations that a heterotrimeric Gα subunit from *N*. *crassa* (GNA-3) is more closely related to proteins from filamentous Ascomycetes and Basidiomycetes than to those from *S*. *cerevisiae* or *S*. *pombe* [69]. We also noted that each of the GNB-1 orthologs from *M*. *circinelloides f*. *lusitanicus* cluster with 1–2 orthologs from *P*. *blakesleeanus*, consistent with an ancient duplication event in an ancestor of these two species and later divergence (Fig 1A). Evidence supporting genome duplication in these species has been previously published [70].

In contrast to the Gβ orthologs, the RACK1 proteins distribute into two major clades, with one corresponding to all of the Ascomycetes (including *N*. *crassa*) and the other containing the Basdiomycetes, Mucoromycetes and Chytridiomycetes (Fig 1B). In the case of the two Mucoromycete species, the RACK1 proteins from each species have the other protein from the same species are their closest neighbor on the tree (Fig 1B). This suggests a more recent gene duplication event for the RACK1 orthologs that occurred after divergence of these two species.

### CPC-2 is a cytoplasmic protein

We utilized two independent methods to assess subcellular localization of CPC-2. First, differential centrifugation was performed on protein extracts from wild type and the fractions subjected to western analysis using antibodies to marker proteins and CPC-2. Since there was no antibody for CPC-2 available prior to our study, we first expressed and purified an MBP-CPC-2 fusion protein from *E*. *coli* and used the protein to produce polyclonal antisera in rabbits (see Materials and methods for details). Tests of the serum showed that it recognized a protein of the predicted molecular mass of CPC-2 (~35 kDa) in whole cell extracts from wild type.

**Fig 2:**
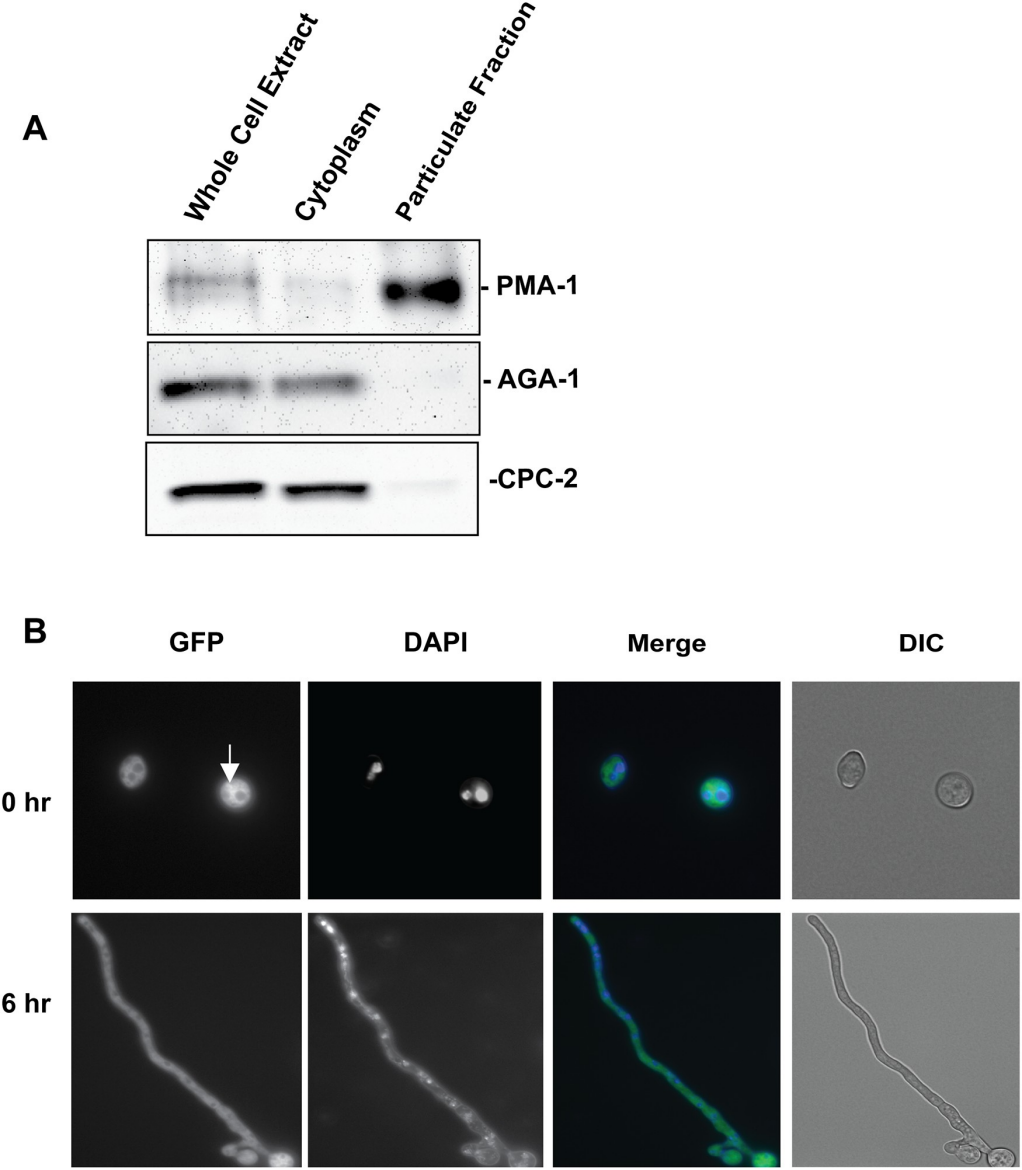
Subcellular localization of CPC-2. **A. Fractionation of CPC-2 during differential centrifugation of cell extracts**. Cytosolic and particulate fractions were isolated from a cell extract of wild-type strain 74-OR23-1VA as described in the Materials and methods. Samples corresponding to the same volume of original cell extract were subjected to SDS-PAGE and western analysis using CPC-2, arginase (AGA; cytosol), and plasma membrane ATPase (PMA-1; plasma membrane) antibodies. The results shown are representative of four biological replicates. **B. Localization of GFP-tagged CPC-2 protein *in vivo***. An aliquot containing 8 x 10^6^ macroconidia from the CPC-2-GFP-9-10 strain was inoculated on VM agarose plates and incubated at 30°C for 0 h and 6 h. Images for the GFP channel were obtained via fluorescence microscopy and also stained with DAPI to visualize the nucleus (see Materials and methods for details). Images for GFP and DAPI were merged using ImageJ (National Institutes of Health, Bethesda, MD). Differential interference contrast (DIC) images were taken to show overall morphology of macroconidia and hyphae. Scale bar = 10 microns.

For the differential centrifugation approach, we generated whole cell extracts, and samples enriched for cytosol and the particulate fraction (membranous organelles and large macromolecular assemblies). Western analysis was performed using antibodies directed against arginase/AGA (cytosolic marker) [67] and the plasma membrane ATPase/PMA-1 (plasma membrane marker) [68], with the results showing good separation of the fractions (Fig 2A). Some contamination of the cytosolic fraction with plasma membranes (but not vice-versa) is evident from the presence of trace amounts of PMA-1 in the cytosol and the absence of the AGA from the particulate fraction. Western analysis using the CPC-2 antibody demonstrated that the great majority of CPC-2 was localized to the cytoplasm, with a small amount in the particulate fraction.

As an alternative method, we implemented fluorescence microscopy to determine the subcellular localization of CPC-2 in a strain expressing a GFP-tagged version of the protein (Fig 2B). The CPC-2-GFP signal was localized in the cytoplasm and excluded from the nucleus (as represented by DAPI staining) in both macroconidia and 6 h germlings (Fig 2B). Thus, both subcellular fractionation and live-cell imaging approaches support a cytoplasmic localization for CPC-2 in *N*. *crassa*.

### Creation of mutants lacking *cpc-2* and G protein subunit genes and analysis of G protein levels in Δ*cpc-2* strains

We have previously demonstrated that components of the G protein signaling pathway are crucial for hyphal growth and asexual and sexual development of *N*. *crassa* [8, 11, 61, 71–73]. In order to explore a possible role for *cpc-2* as a heterotrimeric Gβ gene in *N*. *crassa*, we created strains that could be used for genetic epistasis analysis. We previously employed a similar approach for analysis of genetic relationships between the Gβ *gnb-1* and the three Gα subunit genes [55]. We first purified Δ*cpc-2* homokaryotic knockout mutants from a transformant created during the Neurospora Genome Project [53, 74] (see Materials and methods). We constructed complemented strains carrying the Δ*cpc-2* mutation and a *pan-2* targeted, GFP-tagged version of the *cpc-2*^+^ gene *in trans* (see Materials and methods and Table 1). The complemented strains exhibited significant complementation of several phenotypes, including hyphal growth rate (S2 Fig) and partial complementation of aerial hyphae height (S2 Fig). We used sexual crosses or transformation to generate deletion mutants lacking *cpc-2* alone or in combination with mutations in the three Gα genes or the Gβ, *gnb-1*. We also constructed Δ*cpc-2* strains expressing GTPase-deficient, constitutively activated Gα alleles (*gna-1*^Q204L^, *gna-2*^Q205L^ or *gna-3*^Q208L^; see Materials and methods and Table 1).

**Fig 3:**
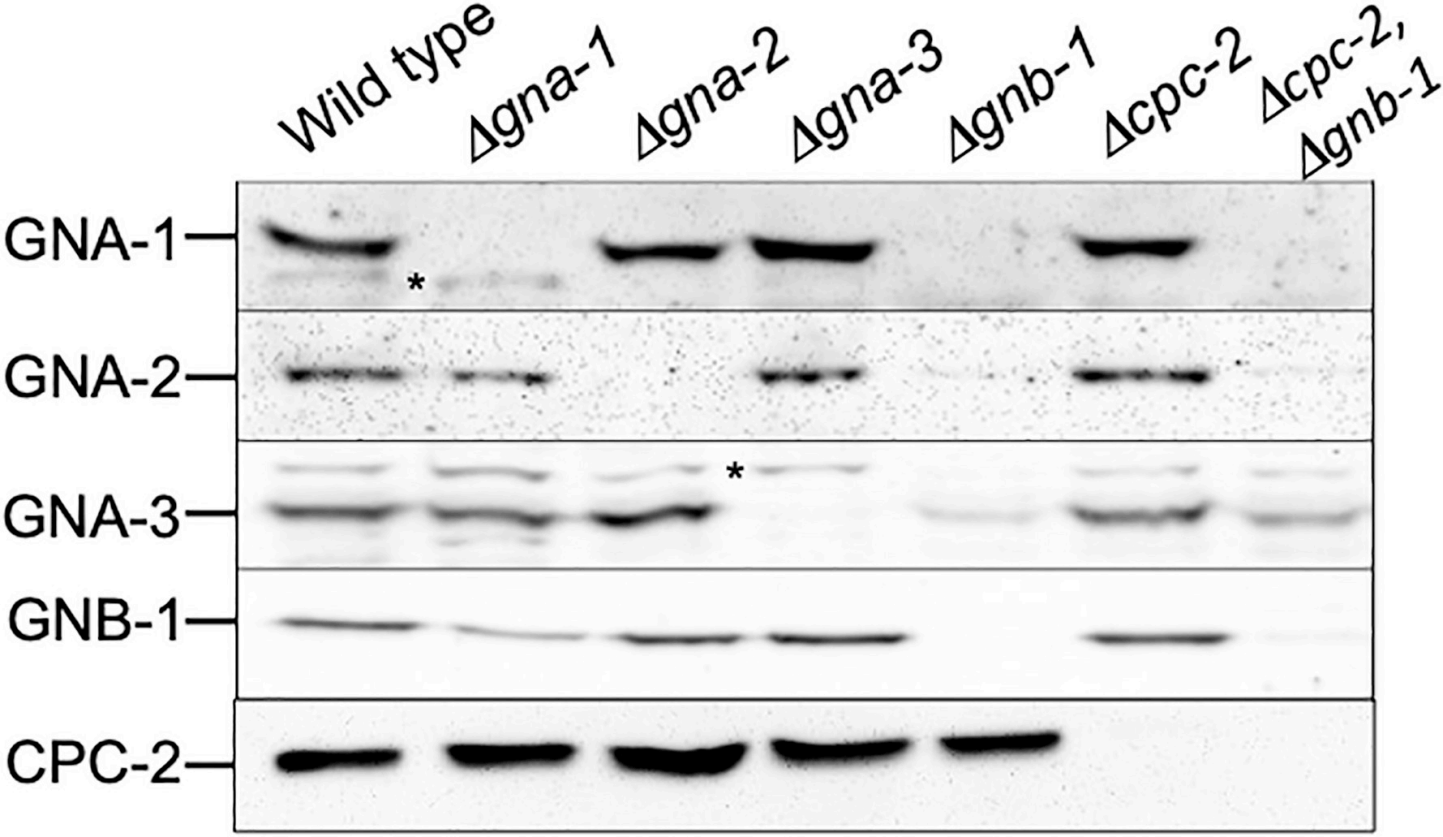
Levels of CPC-2 and other G protein subunits in different strain backgrounds. For detection of GNA-1, GNA-2, GNA-3 and GNB-1, differential centrifugation was used to isolate the particulate fraction from whole cell protein extracts of the indicated strains. Samples were subjected to SDS-PAGE and western blots prepared. Blots were reacted with antiserum for GNA-1, GNA-2, GNA-3 or GNB-1. For detection of CPC-2, protein from whole cell extracts was used to prepare western blots. Blots were reacted with polyclonal antiserum raised against a MBP-CPC-2 fusion protein purified from *E*. *coli*. The results shown are representative of three biological replicates. The migration position of each protein is shown along the right side of the panel.

We have previously shown that, depending on the growth conditions, loss of the Gβ subunit *gnb-1* leads to lower levels of one or all three Gα proteins in *N*. *crassa* [55, 61, 64]. The exact mechanism underlying this regulation is unknown, but appears to be post-transcriptional, as Gα mRNA levels are normal in Δ*gnb-1* mutants [61, 64]. Therefore, prior to initiating genetic epistasis experiments with *cpc-2*, we utilized western blot analysis with protein-specific antisera to check levels of G protein subunits in the Δ*cpc-2* mutant (Fig 3). The results demonstrate that in contrast to *gnb-1*, loss of *cpc-2* does not greatly influence levels of the three Gα proteins or GNB-1 (Fig 3; compare wild type and Δ*cpc-2* lanes). However, Gα protein levels are still reduced when *gnb-1* is mutated in the Δ*cpc-2* background (Fig 3). We also consistently noted an increased level of GNA-3 in the Δ*cpc-2* Δ*gnb-1* double mutant vs. the Δ*gnb-1* single mutant, suggesting that loss of *cpc-2* partially reverses the effect of the Δ*gnb-1* mutation. The observation that the Δ*cpc-2* single mutant has normal levels of G protein subunits greatly streamlines interpretation of genetic epistasis experiments using *cpc-2*.

We next wanted to determine whether loss of any of the G protein subunits affects CPC-2 protein levels. Because CPC-2 is a cytoplasmic protein (Fig 2), we used protein from whole cell extracts for western analysis using the CPC-2 antiserum (Fig 3). The results demonstrated that CPC-2 protein levels were relatively normal in the G protein single mutants (Fig 3). Thus, similar to the situation with GNB-1 levels in the Δ*cpc-2* strain, CPC-2 levels are not affected by loss of *gnb-1*; the two predicted Gβ proteins are independent of one another in this regard. Our findings suggest that if CPC-2 does operate as a Gβ subunit, it does not share all functions with GNB-1 in *N*. *crassa*.

### *cpc-2* is epistatic to *gna-2* during regulation of basal hyphal growth rate

*N*. *crassa* grows by elongation, branching and fusion of hyphae, eventually forming a network structure called the mycelium (rev. in [75]. From this mycelium, aerial hyphae grow upward and spore-forming structures (macroconidiophores) are elaborated from their tips. Formation of cross-walls and constriction of macroconidiophores leads to formation of the mature multinucleated asexual spores, macroconidia. Macroconidia are disseminated in nature by wind currents, enabling the fungus to colonize new areas. When in the presence of water and suitable nutrients, macroconidia germinate to form a hyphal tube, which then begins the growth program described above [75].

We began our genetic epistasis analysis by investigating the set of mutants for defects in basal hyphae extension rate, using macroconidia to inoculate race tubes (see Materials and methods). The results from genetic epistasis analysis were interpreted as reported previously [55]: If the phenotype of the Δ*cpc-2*, ΔGα double mutant resembles the phenotype of the ΔGα mutant, and if the mutationally activated Gα allele bypasses the phenotype of Δ*cpc-2*, then the Gα gene is epistatic to (implied downstream) to *cpc-2*. If the opposite is true, then *cpc-2* is epistatic to the Gα gene. If contradicting results are seen, this is interpreted as the two genes being partially or completely independent in regulation of the phenotype being assessed.

**Fig 4:**
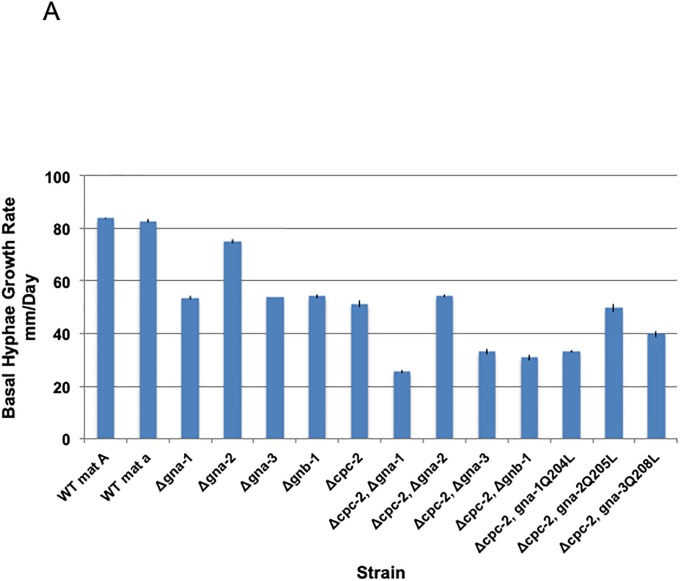
Growth rate of basal hyphae. VM agar race tubes were inoculated with the indicated genotypes, incubated at 25°C and marked at various times as previously described [65]. Linear growth rates were determined with values (expressed as mm/day) taken from four biological replicates. Strains used were 74-OR23-1VA, ORS-SL6a, 3B10, Δgna2-2476, 3lc2, 42-8-3, Δcpc2#11, C2B1#2-1-1, C2G1#39, C2G2#37, C2G3#1–6, C2G1*#44, C2G2*#4 and C2G3*#1–8 (See Table 1 for genotypes). Error was calculated as the standard error of the mean. ANOVA was performed to identify strains that were significantly different from one another (S1 File).

All of the single gene mutants had a basal hyphal growth rate phenotype (Fig 4, S1 File). In Δ*cpc-2* mutants, the growth rate was 61% of wild type (Fig 4). The findings from ANOVA of the characterized strains revealed several relationships (S1 File). First, *cpc-2* may operate downstream of *gna-2*. Both mutants are significantly different than wild type, the double mutant grows slower than Δ*gna-*2, but slightly faster than Δ*cpc-2*, and mutational activation of *gna-2* (*gna-2*^Q205L^ allele) does not lead to an increase in growth rate in the Δ*cpc-2* background. Second, the Δ*gna-1* and Δ*cpc-2* knockout mutations are synergistic with regards to reduction in growth rate, and mutational activation of *gna-1* does not rescue the Δ*cpc-2* phenotype; in fact, the growth rate is further reduced in the Δ*cpc-2 gna-1*^Q204L^ strain. The same relationships hold between *gna-3* and *cpc-2*. These results suggest that *cpc-2* regulates growth rate using a different pathway than *gna-1* or *gna-3*. Finally, Δ*cpc-2* and Δ*gnb-1* are also synergistic, with the double mutant having a significantly slower growth rate than either single mutant (Fig 4). This suggests that these two Gβ-like genes have some independent functions during regulation of hyphal growth in *N*. *crassa*.

We have previously demonstrated that strains lacking either of the G protein subunit genes *gna-1* and *gna-3*, but not *gna-2* or *gnb-1*, have a defect in germination of macroconidia, an essential step prior to hyphal growth and formation of a colony [66]. Therefore, we explored this phenotype in Δ*cpc-2* mutants, using wild type as a control (S3 Fig). Similar to Δ*gnb-1* mutants, strains lacking *cpc-2* are normal with respect to germination of macroconidia (S3 Fig). Thus, overall colony size of Δ*cpc-2* mutants is compromised by slower extension of basal hyphae, and not by a defect in germination of macroconidia.

### *cpc-2* is epistatic to *gna-2* with regards to aerial hyphae height and Δ*cpc-2* mitigates the increased macroconidia production of Δ*gnb-1* mutants on solid medium

**Fig 5:**
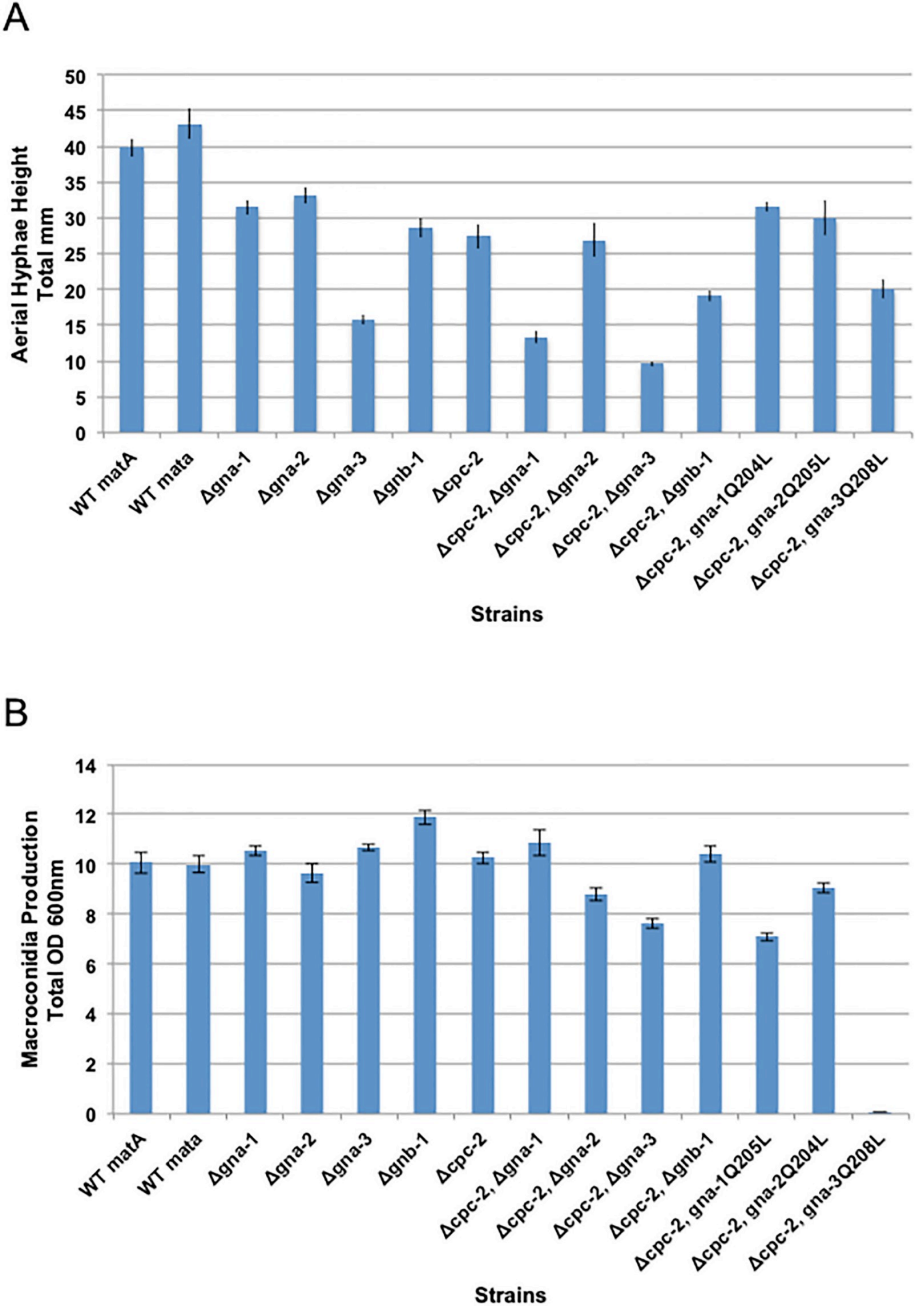
Quantative phenotypes during asexual development. Strains, error calculations and ANOVA were as in Fig 4. **A. Aerial hyphae height**. Culture tubes containing liquid VM medium were inoculated with the indicated strains and incubated statically in the dark for three days at room temperature. The distance grown by the aerial hyphae above the medium interface was then measured. Values (mm) were taken from 12 biological replicates. **B. Macroconidia production**. Macroconidia from the indicated strains were propagated by growth in VM agar culture tubes in the dark at 30°C for four days followed by three days in light at room temperature. Macroconidia were harvested from the cultures and quantitated as described in the Materials and methods. Values represent eight biological replicates.

We next explored epistatic relationships between *cpc-2* and the other genes for two quantitative traits relevant to macroconidiation: aerial hyphae height and macroconidia abundance. Similar to the case for basal hyphae growth rate, all of the single gene deletion mutants had an aerial hyphae height defect (Fig 5A, S1 File). For Δ*cpc-2*, aerial hyphae heights were 69% of wild type (Fig 5A).

ANOVA of the strain set produced results similar to those noted for basal hyphae, above (S1 File). The aerial hyphae height of the Δ*cpc-2* and Δ*cpc-2* Δ*gna-2* double mutants is significantly less than that of the Δ*gna-*2 single mutant and the Δ*cpc-2 gna-2*^Q205L^ strain is similar to the Δ*cpc-2* single mutant (Fig 5A). This result is consistent with *cpc-2* functioning downstream of *gna-2* to control aerial hyphae height. In contrast, *gna-1* and *cpc-2* appear to be independent; the double mutant has shorter aerial hyphae than either single mutant and introduction of *gna-1*^Q204L^ does not rescue the aerial hyphae defect of Δ*cpc-2* (Fig 5A). Δ*gna-3* mutants are shorter than Δ*cpc-2* and the double mutant is similar to Δ*gna-3* (Fig 5A). However, the finding that aerial hyphae height is not rescued by the *gna-3*^Q208L^ allele in the Δ*cpc-2* background (S1 File) supports independent regulation by these two subunits. Δ*cpc-2* and Δ*gnb-1* mutants have similar aerial hyphae height and the double mutant is shorter (Fig 5A; S1 File). As observed for regulation of basal hyphal growth, this finding supports independent signaling by *cpc-2* and *gnb-1* during control of aerial hyphae height in *N*. *crassa*.

Quantitative analysis of macroconidia production in agar slants did not reveal a phenotype for Δ*cpc-2* mutants (Fig 5B, S1 File). In fact, of the single mutants analyzed, only Δ*gnb-1* possessed a phenotype (greater conidia production; Fig 5B) and the phenotype of the Δ*cpc-2* Δ*gnb-1* double mutant was similar to that of Δ*cpc-2* (like wild type). This suggests that loss of *cpc-2* mitigates the overproduction of conidia observed in the Δ*gnb-1* mutant, and that *cpc-2* is epistatic to *gnb-1*. For the Gα subunit double mutants, Δ*cpc-2* Δ*gna-3* produces fewer conidia than either single mutant and differentiation of macroconidia is nearly halted in the Δ*cpc-2 gna-3*^Q208L^ strain (Fig 5B). These results support independence of *cpc-2* and *gna-3* during regulation of macroconidiation. A similar situation exists for *gna-2*, as the Δ*cpc-2* Δ*gna-2* double mutant and the Δ*cpc-2 gna-2*^Q205L^ strain produce fewer conidia than either single mutant (Fig 5B). With *gna-1*, the double mutant is similar to the single mutants, but the Δ*cpc-2 gna-1*^Q204L^ mutant produces less macroconidia, consistent with independence (Fig 5B). The results from analysis of strains carrying the three mutationally activated Gα alleles suggest that all three Gα proteins inhibit macroconidiation when locked in the GTP-bound form.

### Δ*cpc-2* mutants produce macroconidia in submerged cultures

Wild-type *N*. *crassa* strains do not differentiate macroconidia while growing in shaken submerged culture unless subjected to heat shock, nitrogen or carbon starvation [76–80]. We have previously demonstrated that loss of the G protein subunits *gna-3*, *gnb-1* and *gng-1* leads to macroconidiation in submerged culture under all conditions [61, 64, 69], while Δ*gna-1* mutants only form macroconidia at high inoculation cell density (≥3x10^6^/ml) in liquid culture [81].

**Fig 6:**
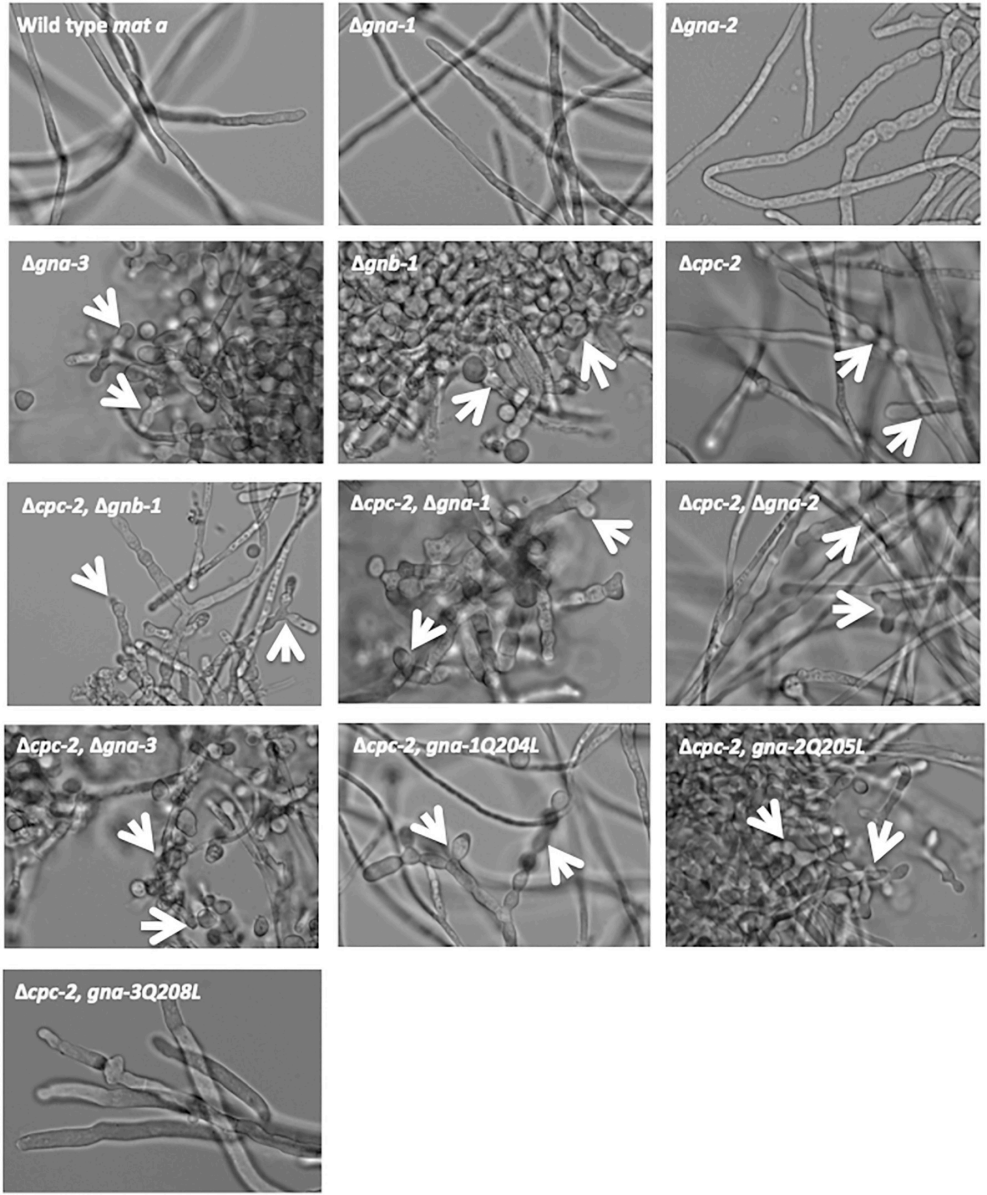
Morphology in submerged culture. Macroconidia isolated from the strains used in Fig 4 were inoculated at a concentration of 1x10^6^ macroconidia/ml and cultured in VM liquid medium for 16 h with shaking at 200 rpm in the dark at 30°C. In the case of strain C2G3*#1–8 (Δ*cpc-2 gna-3*^Q208L^), a small volume of aerial hyphae was used to inoculate cultures, as this strain does not produce a significant amount of macroconidia. A sample of each culture was imaged at 40x magnification using DIC (see Materials and methods). Examples of conidiophores and/or free macroconidia are indicated by the white arrows.

Based on the precedent that the Gβ gene *gnb-1* is a negative regulator of macroconidiation in submerged cultures, we analyzed our group of strains for phenotypes at a low inoculation density (1x10^6^/ml). Similar to previous findings, wild type and Δ*gna-2* mutants do not produce macroconidiophores in submerged culture, while single mutants lacking *gna-3*, *gnb-1* and *gng-1* all produce abundant macroconidiophores (Fig 6). Rare macroconidiophores could also be observed in the Δ*gna-1* strain. We also noted that Δ*cpc-2* knockout mutants produce macroconidiophores in submerged culture (Fig 6).

Double mutants Δ*cpc-2* Δ*gna-1*, Δ*cpc-2* Δ*gna-2*, Δ*cpc-2* Δ*gna-3* and Δ*cpc-2* Δ*gnb-1* all produce conidia in submerged culture. In all four cases, loss of *cpc-2* either leads to submerged conidiation or intensifies the conidiation phenotype of the G protein subunit mutants and the *Δgna-3* Δ*cpc-2* double mutant cultures are mostly conidia (Fig 6). Interestingly, introduction of mutationally activated *gna-3* corrects the submerged conidiation phenotype of Δ*cpc-2*, while the corresponding activated alleles of *gna-1* or *gna-2* do not (Fig 6). This result suggests that GNA-3 may operate downstream of CPC-2, but also has a CPC-2 independent function in controlling submerged conidiation.

### Constitutive activation of Gα subunits does not restore female fertility to the Δ*cpc-2* mutant

*N*. *crassa* is a heterothallic organism, meaning that a given strain has one of two different mating type genes present at a single genomic locus (idiomorphs; *mat A* or *mat a*) [82, 83]. Upon nitrogen limitation, *N*. *crassa* forms protoperithecia (the female reproductive structures) [75, 84]. In the presence of a male cell (usually conidia) of the opposite mating type, pheromone detection results in chemotropic growth of specialized hyphae called trichogynes from the protoperithecium. The fruiting body, or perithecium, is then formed and contains asci, each with eight haploid spores (ascospores). Upon maturation, ascospores are ejected from the tips (beaks) of perithecia, in the direction of light. Under laboratory conditions, protoperithecial development can be induced using Synthetic Crossing Medium (SCM), and progeny are obtained from sexual crosses approximately 2–3 weeks post-fertilization [75, 84].

Our previous work showed that the mutationally activated *gna-1*^Q204L^, *gna-2* ^Q205L^ and *gna-3*^Q208L^ alleles were not able to restore fertility to the Δ*gnb-1* mutant. In fact, introduction of *gna-3*^Q208L^ resulted in complete inhibition of protoperithecial development, a phenotype that was more severe than that of the Δ*gnb-1* mutant [55]. Δ*gnb-1* ΔGα double mutant strains resemble the Δ*gnb-1* mutant, in that they form protoperithecia, but no perithecia after fertilization [55].

**Fig 7:**
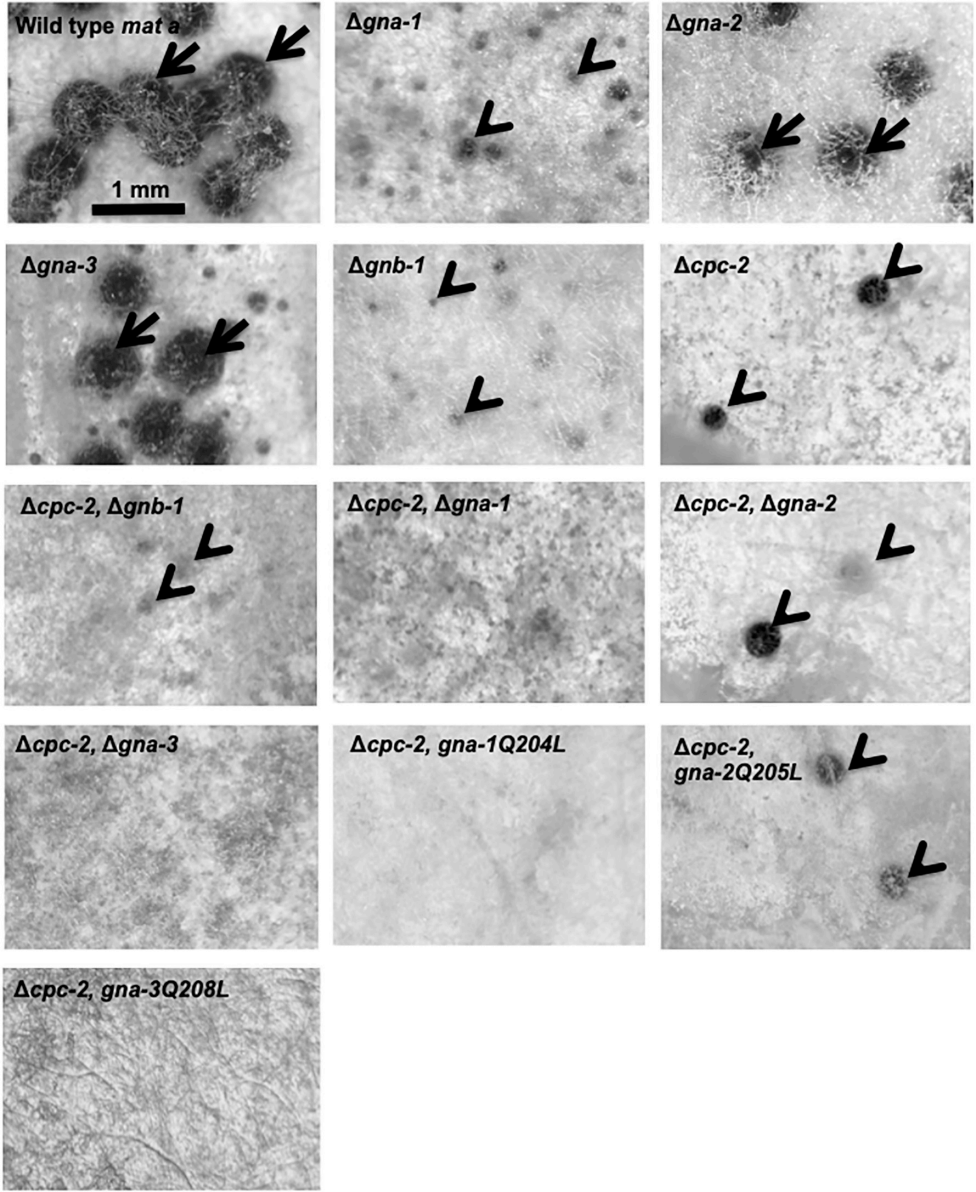
Phenotypes during the sexual cycle. Strains were ORS-SL6a, 3B10, Δgna2-2476, 3lc2, 42-8-3, Δcpc2#11, C2B1#2-1-1, C2G1#39, C2G2#37, C2G3#1–6, C2G1*#44, C2G2*#4 and C2G3*#1–8 (See Table 1 for genotypes). Macroconidia or hyphae from strains were inoculated onto SCM plates and incubated in constant light at room temperature for 7 days. At that time, half of each plate was inoculated with either macroconidia (males) of opposite mating type or water (control). Males were from wild-type strains 74-OR23-1VA (*mat A*) or ORS-SL6a (*mat a*). Incubation was continued under the same conditions for an additional 7 days. The fertilized side of each plate was then photographed using a Leica S8APO stereomicroscope with a DFC280 camera (Leica Microsystems, Buffalo Grove, IL USA). Examples of protoperithecia or submerged, aberrant perithecia are indicated by the black arrowheads, while mature perithecia are shown by the black arrows.

Muller et al. [33] previously reported that *cpc-2* point mutants do not produce protoperithecia and are thus female-sterile. In contrast, our results with the Δ*cpc-2* knockout mutant indicate some protoperithecia are present, as the cultures produce rare perithecia after fertilization that are mostly submerged in the agar (Fig 7). This phenotype is distinct from that of Δ*gna-2* and Δ*gna-3* strains that produce perithecia similar to wild type and from Δ*gna-1* and Δ*gnb-1* mutants that do not form perithecia after fertilization (Fig 7).

Inspection of double mutants revealed that Δ*cpc-2* Δ*gna-1* strains do not produce visible protoperithecia, perithecia or ascospores (Fig 7), a more severe phenotype than either single mutant. In contrast, Δ*cpc-2* Δ*gna-2* mutants resemble Δ*cpc-2* single mutants. Δ*cpc-2* Δ*gna-3* strains exhibit a variable phenotype, with either small, submerged perithecia or no visible perithecia (Fig 7), and no ascospores. Mutational activation of either *gna-1* or *gna-3* in the Δ*cpc-2* background leads to no visible protoperithecia, perithecia or ascospores, while activation of *gna-2* results in the Δ*cpc-2* phenotype (Fig 7). These results are consistent with synergy between *cpc-2* and *gna-1* and *gna-3*. The phenotype of Δ*cpc-2 gna-3*^Q208L^ and Δ*gnb-1 gna-3*^Q208L^ strains are similar [55], suggesting a common mode of action for *gna-3*^Q208L^ and/or interaction between GNA-3 and the two candidate Gβ proteins. In contrast, the different results observed after introduction of *gna-1*^Q204L^ into the two mutants hints at a different role for CPC-2 vs. GNB-1 during regulation of female fertility.

As noted previously, the Δ*gnb-1* strain forms small, aberrant protoperithecia, but no perithecia, upon fertilization [61] (Fig 7). In contrast, similar to Δ*gnb-1* single mutants, Δ*cpc-2* Δ*gnb-1* double mutants do not produce perithecia (Fig 7). This result suggests that *gnb-1* is epistatic to *cpc-2* during sexual development.

## Discussion

**Fig 8:**
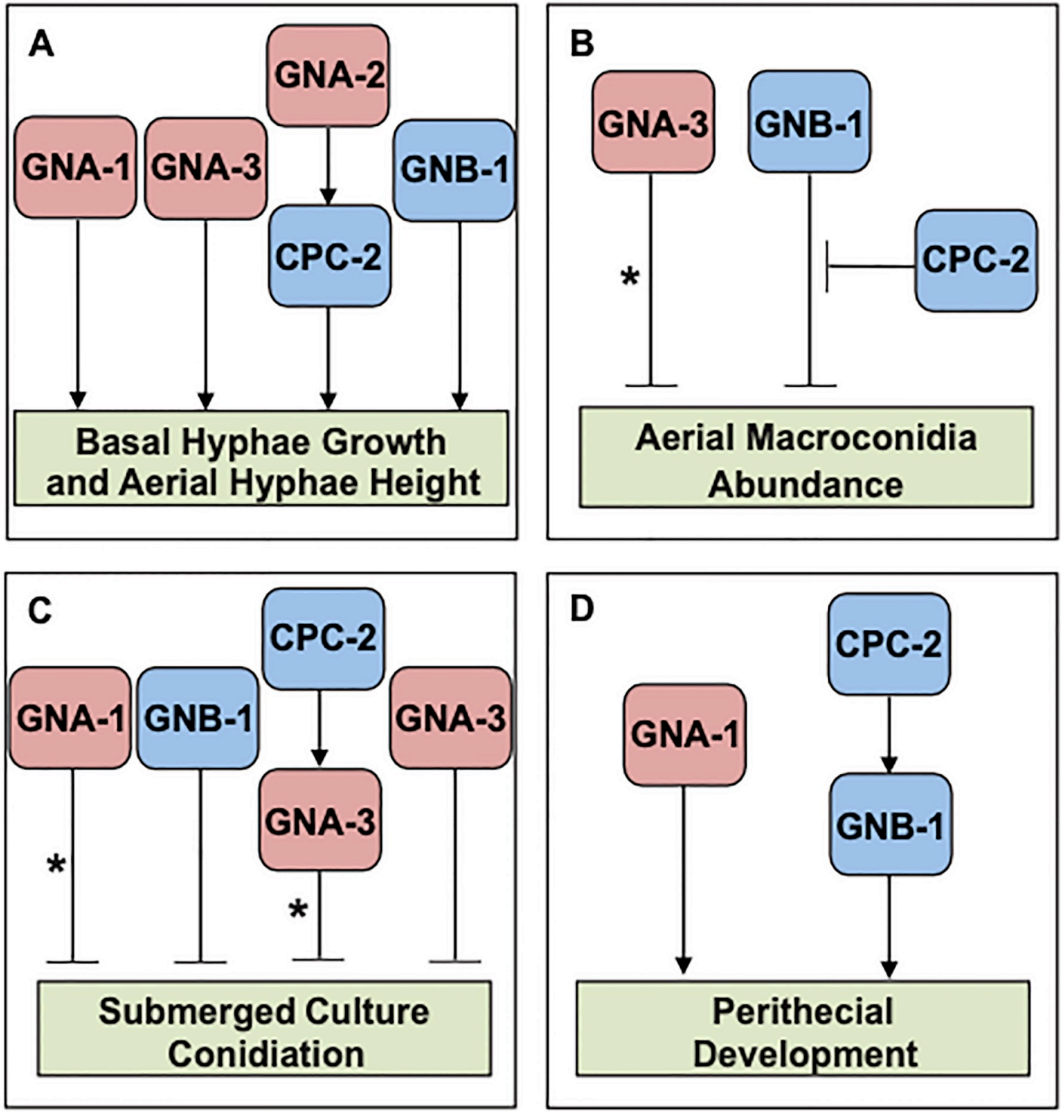
Models for interactions between CPC-2 and G protein subunits in *N*. *crassa*. The three Gα proteins are colored red, while the two predicted Gβ proteins are blue in the various panels. **A. Basal hyphae growth and aerial hyphae height**. GNA-2 operates upstream of CPC-2 to positively modulate basal hyphae growth rate and aerial hyphae height. GNA-1, GNA-3 and GNB-1 act independently of CPC-2 to regulate this trait. **B. Aerial macroconidia abundance**. GNB-1 is a negative regulator of macroconidia abundance in agar cultures and loss of *cpc-2* suppresses this effect. The asterisk indicates that GNA-3 only influences macroconidiation when mutationally activated. **C. Submerged culture conidiation**. CPC-2, GNA-1, GNA-3 and GNB-1 are negative regulators of conidiation in liquid submerged cultures. The asterisk on the GNA-1 arrow denotes the cell density dependence of the GNA-1 effect. The asterisk on the GNA-3 arrow indicates that GNA-3 may function with CPC-2 or independently, as Δ*gna-3* Δ*cpc-2* mutants have a more severe phenotype than the single mutants, but mutational activation of *gna-3* corrects the phenotype of the Δ*cpc-2* mutant. **D. Perithecial development**. GNB-1 functions downstream of CPC-2 to regulate perithecial development. The action of GNA-1 to control perithecial development is independent of CPC-2.

The *N*. *crassa cpc-2* gene is not essential and the encoded protein is similar to other RACK1 homologs in fungi. Genetic epistasis between *cpc-2* and components of the G protein pathway was performed using double deletion mutants and strains containing Gα activated alleles (see model in Fig 8). The results revealed genetic relationships between *cpc-2* and *gna-2* during growth of basal and aerial hyphae, *cpc-2* and *gna-3* during growth in submerged cultures and *cpc-2* and *gnb-1* in regulation of sexual development. In the cases of basal and aerial hyphae growth, the epistatic relationships suggest that CPC-2 operates downstream of the Gα protein, implying a tethering function for the Gα in regulation of CPC-2. However, the GNA-3 Gα acts downstream of CPC-2 during submerged culture conidiation, suggesting that the RACK1 protein is holding GNA-3 inactive. CPC-2 appears to operate upstream of the Gβ GNB-1 during sexual development and to act in an antagonistic function during production of macroconidia in agar cultures.

Our investigation of epistasis between the three Gα genes and *gnb-1* and *cpc-2* revealed some interesting parallels. As mentioned above, the results from the current study suggest that *gna-3* is at least partially epistatic to *cpc-2* during control of appropriate conidiation in submerged cultures. This is similar to the earlier relationship observed for *gnb-1* and *gna-3* for this same phenotype, with *gna-3* epistatic to *gnb-1* [55]. The other two Gα subunits are independent of both *cpc-2* (this study) and *gnb-1* [55] during the regulation of this trait. This indicates that *cpc-2*, like *gnb-1*, is a negative regulator of conidiation in submerged culture, and that only activation of *gna-3* offers a genetic bypass mechanism to restore normal hyphal growth. Our previous results from epistasis studies of aerial hyphae height demonstrated that *gnb-1* is epistatic to both *gna-2* and *gna-3* and independent of *gna-1* [55]. Together with the current study, the findings are consistent with a model in which the Gβ gene lies downstream of the Gα gene(s) and that *gna-1* is independent of both *gnb-1* and *cpc-2* during aerial hyphae elongation. However, any conclusions based on Δ*cpc-2* Δ*gnb-1* double mutants need to be tempered, as loss of *gnb-1* leads to decreased levels of the three Gα proteins in all genetic backgrounds tested.

It is intriguing that the Δ*cpc-2* Δ*gnb-1* double mutants have higher levels of GNA-3 protein than Δ*gnb-1* single mutants (but still less than in wild type; Fig 3A). This finding suggests that loss of *cpc-2* partially mitigates the effects of the Δ*gnb-1* mutation. In a canonical model for G protein signaling, GNB-1 would function as a GDI for GNA-3 and loss of GNB-1 might lead to misfolding and/or proteolysis of GNA-3. Mutation of *cpc-2* partially counteracts this effect, suggesting that CPC-2 participates in the pathway leading to decreased levels of GNA-3 protein. Furthermore, the finding that Gα single mutants have slower basal hyphae growth rates than wild type and that loss of *gnb-1* leads to lower levels of Gα proteins supports a possible tethering function for GNB-1 during hyphal growth. Loss of one Gα protein could free more GNB-1 to bind the other Gα subunits, potentially inhibiting them from serving as positive regulators of basal hyphae growth rate. Along these lines, it has been demonstrated in *S*. *cerevisiae* that levels of the Gα protein Gpa1p are regulated by ubiquitin-mediated proteolysis, and it has been proposed that this is a mechanism used to modulate levels of the active, free Gβγ dimer during mating [85, 86].

Analysis of the sexual cycle demonstrated that the Δ*cpc-2* forms rare protoperithecia and perithecia and is therefore female sterile. In contrast, mutants lacking other components of the cross pathway control network (*cpc-1* and *cpc-3*) have normal sexual cycles [87, 88]. This indicates that the sexual cycle defect of Δ*cpc-2* mutants is not solely due to a defect in the response to amino acid limitation. However, there is a possibility that the two processes may be linked. It has been reported in *A*. *nidulans* that amino acid limitation arrests sexual development [28]. Furthermore, loss of the RACK1/*cpc-2* homolog *cpcB* or overexpression of the *cpc-1* homolog *cpcA* also block sexual development, supporting a link between the sexual cycle program and the network that regulates amino acid biosynthesis [28].

Attempts to detect an interaction between CPC-2 and other G protein subunits in *N*. *crassa* using the yeast two-hybrid assay were unsuccessful. Presumably due to the large number of binding partners, the difficulty in solubilizing peripheral membrane proteins such as Gα subunits, and protein folding concerns with heterologously expressed proteins, we were also unable to achieve co-immunoprecipitation between CPC-2 and GNB-1 or any of the three Gα proteins using cell extracts or proteins expressed and purified from *E*. *coli*. A similar result has been reported for the RACK1 homolog RAK1 in *U*. *maydis* [30]. Knowledge of the interactions between RACK1 and G protein subunits is important for full understanding of the biology of G protein signaling. Therefore, experiments such as these and others that investigate the detailed mechanistic wiring that connects CPC-2 to heterotrimeric G protein signaling will be the focus of future work.

## Supporting information

S1 Fig Strain genotyping using PCR. Strains created in this study were checked for proper integration of the DNA construct at the correct locus via diagnostic Polymerase Chain Reactions (PCRs). Genomic DNA was isolated from the indicated genotypes and used in PCRs with the indicated primers. After electrophoresis, agarose gels were stained using ethidium bromide and imaged. The strains used were 74-OR23-1VA (Wild Type), 3B10 (Δ*gna-1*), Δgna2-2477 *(*Δ*gna-2*), 31c2 (Δ*gna-3*), 42-8-3 (Δ*gnb-1*), Δcpc2#11 (Δ*cpc-2*), Δcpc2#6 (Δ*cpc-2* Δ*mus-52*), C2G1*#44 (Δ*cpc-2 gna-1*^*Q204L*^), C2G2*#4 (Δ*cpc-2 gna-2*^*Q205L*^), C2G3*#1–8 (Δ*cpc-2 gna-3*^*Q208L*^), C2G1#39 (Δ*cpc-2* Δ*gna-1*), C2G2#37 (Δ*cpc-2* Δ*gna-2*), C2G3#1–6 (Δ*cpc-2* Δ*gna-3*), C2B1#2-1-1 (Δ*cpc-2* Δ*gnb-1*), CPC-2-GFP-9-10 (*cpc-2*::*GFP*), and CPC-2-GFP-13.2 (Δ*cpc-2 cpc-2*::*GFP*). **A. Δ*gna-1***. Primers #4 and #14 were used to amplify a 1.45 kb band corresponding to the Δ*gna-1* deletion from the indicated strains. **B. Δ*gna-2***. Primers #7 and #14 were used to amplify a 1.3 kb band corresponding to the Δ*gna-2* deletion from the indicated strains. **C. Δ*gna-3***. Primers #9 and #13 were used to amplify a 1 kb band corresponding to the Δ*gna-3* deletion from the indicated strains. **D. Δ*gnb-1***. Primers #21 and #22 were used to amplify a 1.3 kb band corresponding to the Δ*gnb-1* deletion from the indicated strains. **E. *ccg-1* promoter-*cpc-2* ORF region**. Primers #23 and #29 were used to amplify a 1.0 kb band corresponding to the *ccg-1* promoter-*cpc-2* ORF region from the indicated strains. **F. *ccg-1* promoter-*gna-1* ORF region**. Primers #23 and #24 were used to amplify a 1.3 kb band corresponding to the *ccg-1* promoter-*gna-1* ORF region from indicated strains. **G. *ccg-1* promoter-*gna-2* ORF region**. Primers #23 and #25 were used to amplify a 1.4 kb band corresponding to the *ccg-1* promoter-*gna-2* ORF region from indicated strains. **H. *ccg-1* promoter-*gna-3* ORF region**. Primers #23 and #26 were used to amplify a 1.5 kb band corresponding to the *ccg-1* promoter-*gna-3* ORF region from indicated strains. **I. Δ*cpc-2***. Primers #1 and #14 were used to amplify a 1.1 kb band corresponding to the Δ*cpc-2* deletion from the indicated strains.
(TIFF)

S2 Fig Analysis of growth rate and asexual development in a complemented strain. Δ*cpc-2* complemented strain CPC-2-GFP-13.2 was compared to wild type (WT matA) and Δ*cpc-2* strain Δcpc2#11 with respect to growth rate of basal hyphae (top; four replicates) and aerial hyphae height (bottom; 12 replicates) on VM medium supplemented with 10 μg/ml pantothenate. Error is indicated as the standard error of the mean. *** p value <0.001 relative to wild type.
(TIFF)

S3 Fig Germination of macroconidia. Macroconidia were harvested as described in [59]. An aliquot containing 8×10^6^ macroconidia was spread on a VM agar plate (100mm plate containing 10 ml agar medium) and spore germination monitored microscopically at 30°C over the indicated times. DIC (differential interference contrast) micrograph images were obtained using an Olympus IX71 microscope with a QIClick digital CCD camera and analyzed using Metamorph software. Strains used were wild type, Δ*cpc-2* and Δ*gnb-1*.
(TIFF)

S1 File ANOVA analysis. Data for basal hyphae growth rate, aerial hyphae height and conidia abundance were analyzed for statistical significance using an Ordinary One-Way ANOVA test with GraphPad Prism 6.0. The p-value cutoff was set to 0.05, confidence intervals were 95% and pair-wise comparisons were made.
(XLSX)

S1 Raw Images Raw images used for western blots and agarose gels in figures. All images were captured using a CCD camera. **A. Western blot used to generate the top panel of** Fig 2A. Cytosolic and particulate fractions were isolated from a protein extract of wild-type strain 74-OR23-1VA as described in the Materials and methods. Samples corresponding to the same volume of original cell extract were subjected to SDS-PAGE and western analysis using plasma membrane ATPase (PMA-1; plasma membrane) antibody. Positions of molecular weight markers are indicated along the right side of the blot. The western blot was treated with chemiluminescence solution and exposed for 5 min. The image was flipped horizontally and darkened for the final figure. The results shown are representative of four biological replicates. **B. Western blot used to generate the middle panel of** Fig 2A. Cytosolic and particulate fractions were isolated from a protein extract of wild-type strain 74-OR23-1VA as described in the Materials and methods. Samples corresponding to the same volume of original cell extract were subjected to SDS-PAGE and western analysis using arginase (AGA; cytosol) antibody. Positions of molecular weight markers are indicated along the right side of the blot. The western blot was treated with chemiluminescence solution and exposed for 1 min. The image was flipped horizontally and darkened for the final figure. The results shown are representative of four biological replicates. **C. Western blot used to generate the bottom panel of** Fig 2A. Cytosolic and particulate fractions were isolated from a protein extract of wild-type strain 74-OR23-1VA as described in the Materials and methods. Samples corresponding to the same volume of original cell extract were subjected to SDS-PAGE and western analysis using CPC-2 antibody. Positions of molecular weight markers are indicated along the right side of the blot. The western blot was treated with chemiluminescence solution and exposed for 1 min. The image was flipped horizontally and darkened for the final figure. The results shown are representative of four biological replicates. **D. Western blot used to generate the top panel of** Fig 3. For detection of GNA-1, differential centrifugation was used to isolate the particulate fraction from whole cell protein extracts of the indicated strains. Samples were subjected to SDS-PAGE and western blots prepared. Blots were reacted with antiserum for GNA-1. Positions of molecular weight markers are indicated on the left side of the blot. The western blot was treated with chemiluminescence solution and exposed for 1 min. The image was flipped horizontally for Fig 3. The results shown are representative of three biological replicates. **E. Western blot used to generate the second panel of** Fig 3. For detection of GNA-2, differential centrifugation was used to isolate the particulate fraction from whole cell protein extracts of the indicated strains. Samples were subjected to SDS-PAGE and western blots prepared. Blots were reacted with antiserum for GNA-2. Positions of molecular weight markers are indicated on the left side of the blot. The western blot was treated with chemiluminescence solution and exposed for 1 min. The image was flipped horizontally for Fig 3. The results shown are representative of three biological replicates. **F. Western blot used to generate the third panel of** Fig 3. For detection of GNA-3 differential centrifugation was used to isolate the particulate fraction from whole cell protein extracts of the indicated strains. Samples were subjected to SDS-PAGE and western blots prepared. Blots were reacted with antiserum for GNA-3. Positions of molecular weight markers are indicated on the left side of the blot. The western blot was treated with chemiluminescence solution and exposed for 1 min. The results shown are representative of three biological replicates. **G. Western blot used to generate the fourth panel of** Fig 3. For detection of GNB-1, differential centrifugation was used to isolate the particulate fraction from whole cell protein extracts of the indicated strains. Samples were subjected to SDS-PAGE and western blots prepared. Blots were reacted with antiserum for GNB-1. Positions of molecular weight markers are indicated on the left side of the blot. The western blot was treated with chemiluminescence solution and exposed for 1 min. The results shown are representative of three biological replicates. **H. Western blot used to generate the bottom panel of** Fig 3. Protein from whole cell extracts was used to prepare western blots that were then reacted with CPC-2 antibody. Positions of molecular weight markers are indicated along the left side of the blot. The western blot was treated with chemiluminescence solution and exposed for 1 min. The image was darkened for the final figure. The results shown are representative of three biological replicates. **I. Agarose gel used to generate** S1A Fig. Primers #4 and #14 were used to amplify a 1.45 kb band corresponding to the Δ*gna-1* deletion mutation from the indicated strains. Positions of molecular weight markers are indicated on the left side of the gel. The agarose gel was soaked in ethidium bromide and exposed to UV light for 100 ms. **J. Agarose gel used to generate** S1B Fig. Primers #7 and #14 were used to amplify a 1.3 kb band corresponding to the Δ*gna-2* deletion mutation from the indicated strains. Positions of molecular weight markers are indicated on the left side of the gel. The agarose gel was soaked in ethidium bromide and exposed to UV light for 100 ms. **K. Agarose gel used to generate** S1C Fig. Primers #9 and #13 were used to amplify a 1 kb band corresponding to the Δ*gna-3* deletion mutation from the indicated strains. Positions of molecular weight markers are indicated on the left side of the gel. The agarose gel was soaked in ethidium bromide and exposed to UV light for 100 ms. **L. Agarose gel used to generate** S1D Fig. Primers #21 and #22 were used to amplify a 1.3 kb band corresponding to the Δ*gnb-1* deletion mutation from the indicated strains. Positions of molecular weight markers are indicated on the left side of the gel. The agarose gel was soaked in ethidium bromide and exposed to UV light for 100 ms. **M. Agarose gel used to generate** S1E–S1H Fig. Primers #23 and #29 were used to amplify a 1.0 kb band corresponding to the *ccg-1* promoter-*cpc-2* ORF region from the indicated strains. Primers #23 and #24 were used to amplify a 1.3 kb band corresponding to the *ccg-1* promoter-*gna-1* ORF region from indicated strains. Primers #23 and #25 were used to amplify a 1.4 kb band corresponding to the *ccg-1* promoter-*gna-2* ORF region from indicated strains. Primers #23 and #26 were used to amplify a 1.5 kb band corresponding to the *ccg-1* promoter-*gna-3* ORF region from indicated strains. Positions of molecular weight markers are indicated on the left side of the gel. The agarose gel was soaked in ethidium bromide and exposed to UV light for 50 ms. **N. Agarose gel used to generate** S1I Fig. Primers #1 and #14 were used to amplify a 1.1 kb band corresponding to the Δ*cpc-2* deletion mutation from the indicated strains. Positions of molecular weight markers are indicated on the left side of the gel. The agarose gel was soaked in ethidium bromide and exposed to UV light for 100 ms.
(PDF)
