# Supplementary figures and images for: Genetic relationships between the RACK1 homolog cpc-2 and heterotrimeric G protein subunit genes in Neurospora crassa
Source: PLoS One. 2019 Oct 3;14(10):e0223334. doi: 10.1371/journal.pone.0223334 (PMC6776386; doi:10.1371/journal.pone.0223334)

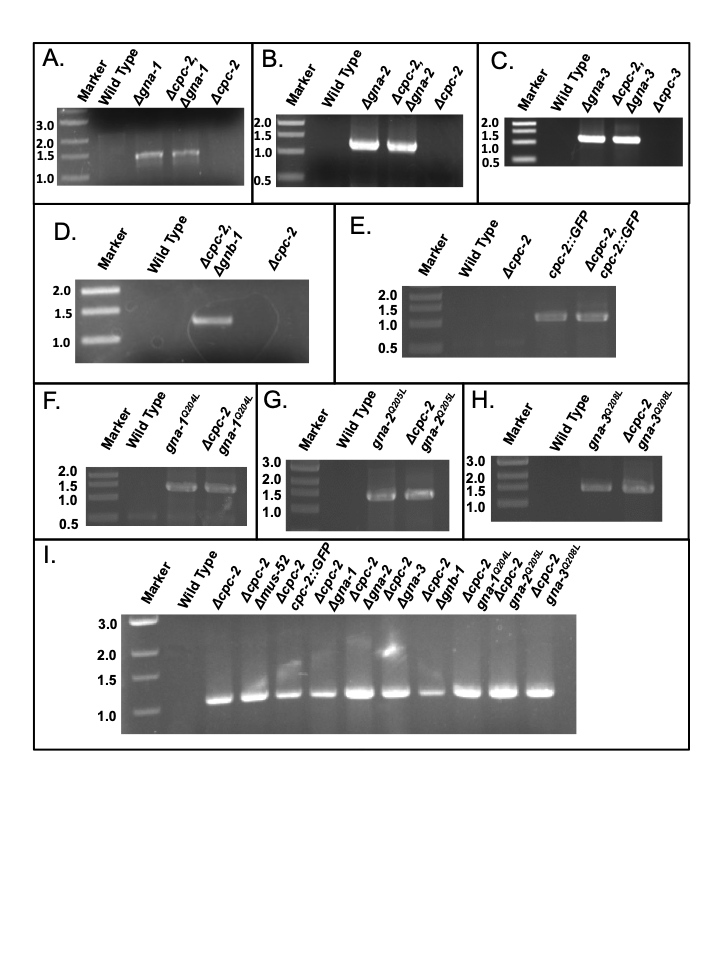

Supplement: S1 Fig — Strains created in this study were checked for proper integration of the DNA construct at the correct locus via diagnostic Polymerase Chain Reactions (PCRs). Genomic DNA was isolated from the indicated genotypes and used in PCRs with the indicated primers. After electrophoresis, agarose gels were stained using ethidium bromide and imaged. The strains used were 74-OR23-1VA (Wild Type), 3B10 (Δgna-1), Δgna2-2477 (Δgna-2), 31c2 (Δgna-3), 42-8-3 (Δgnb-1), Δcpc2#11 (Δcpc-2), Δcpc2#6 (Δcpc-2 Δmus-52), C2G1*#44 (Δcpc-2 gna-1Q204L), C2G2*#4 (Δcpc-2 gna-2Q205L), C2G3*#1–8 (Δcpc-2 gna-3Q208L), C2G1#39 (Δcpc-2 Δgna-1), C2G2#37 (Δcpc-2 Δgna-2), C2G3#1–6 (Δcpc-2 Δgna-3), C2B1#2-1-1 (Δcpc-2 Δgnb-1), CPC-2-GFP-9-10 (cpc-2::GFP), and CPC-2-GFP-13.2 (Δcpc-2 cpc-2::GFP). A. Δgna-1. Primers #4 and #14 were used to amplify a 1.45 kb band corresponding to the Δgna-1 deletion from the indicated strains. B. Δgna-2. Primers #7 and #14 were used to amplify a 1.3 kb band corresponding to the Δgna-2 deletion from the indicated strains. C. Δgna-3. Primers #9 and #13 were used to amplify a 1 kb band corresponding to the Δgna-3 deletion from the indicated strains. D. Δgnb-1. Primers #21 and #22 were used to amplify a 1.3 kb band corresponding to the Δgnb-1 deletion from the indicated strains. E. ccg-1 promoter-cpc-2 ORF region. Primers #23 and #29 were used to amplify a 1.0 kb band corresponding to the ccg-1 promoter-cpc-2 ORF region from the indicated strains. F. ccg-1 promoter-gna-1 ORF region. Primers #23 and #24 were used to amplify a 1.3 kb band corresponding to the ccg-1 promoter-gna-1 ORF region from indicated strains. G. ccg-1 promoter-gna-2 ORF region. Primers #23 and #25 were used to amplify a 1.4 kb band corresponding to the ccg-1 promoter-gna-2 ORF region from indicated strains. H. ccg-1 promoter-gna-3 ORF region. Primers #23 and #26 were used to amplify a 1.5 kb band corresponding to the ccg-1 promoter-gna-3 ORF region from indicated strains. I. Δcpc-2. Primers #1 and #14 were used [file pone.0223334.s001.tiff]

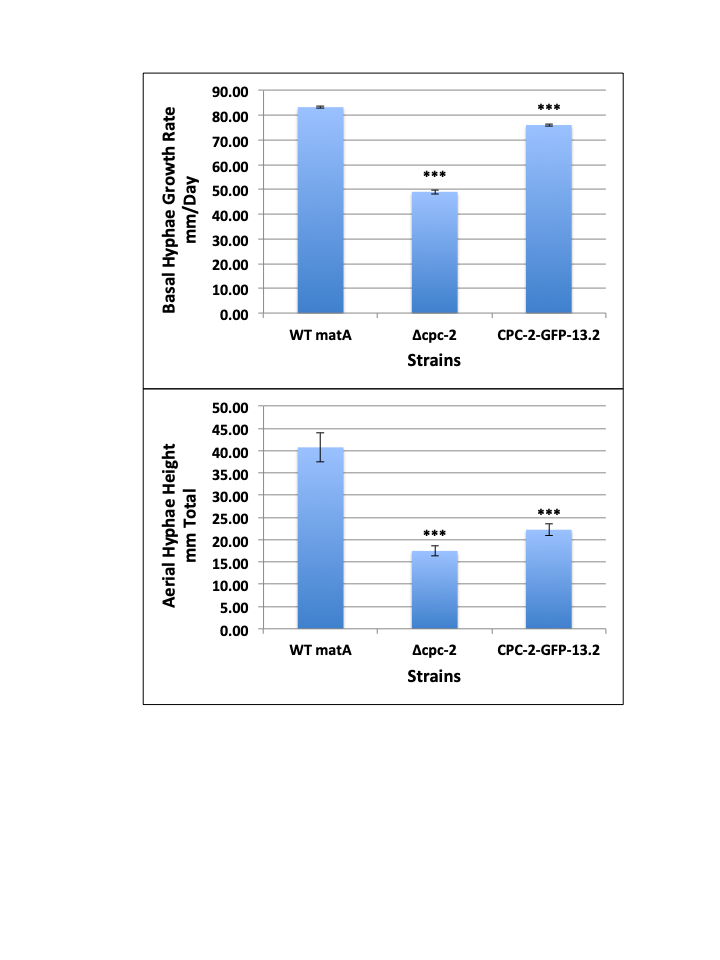

Supplement: S2 Fig — Δcpc-2 complemented strain CPC-2-GFP-13.2 was compared to wild type (WT matA) and Δcpc-2 strain Δcpc2#11 with respect to growth rate of basal hyphae (top; four replicates) and aerial hyphae height (bottom; 12 replicates) on VM medium supplemented with 10 μg/ml pantothenate. Error is indicated as the standard error of the mean. *** p value <0.001 relative to wild type. (TIFF) [file pone.0223334.s002.tiff]

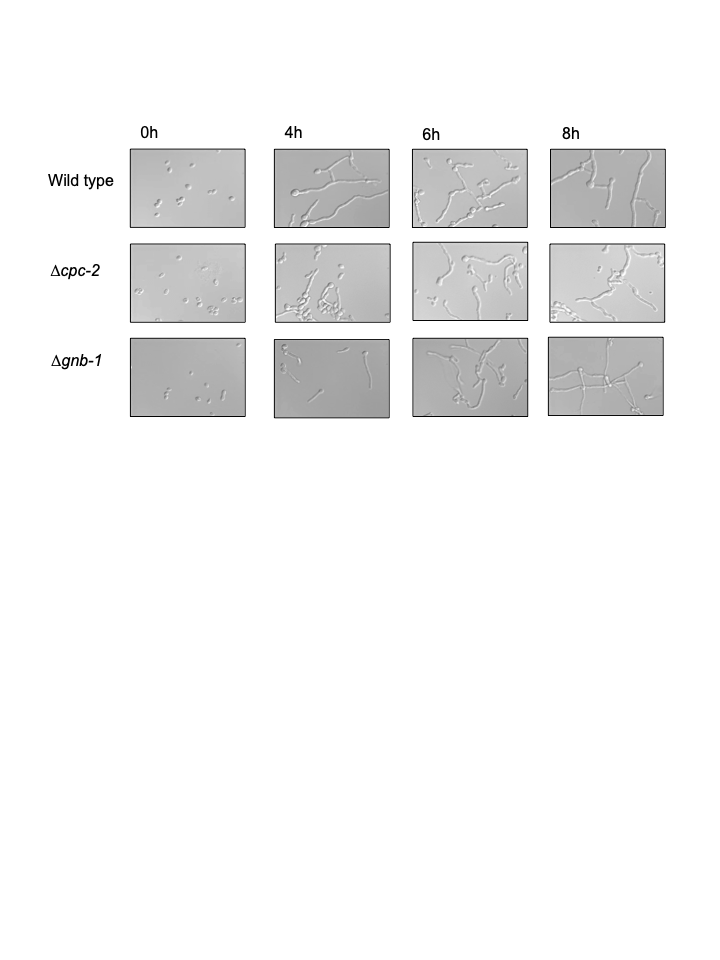

Supplement: S3 Fig — Macroconidia were harvested as described in [59]. An aliquot containing 8×10^6^ macroconidia was spread on a VM agar plate (100mm plate containing 10 ml agar medium) and spore germination monitored microscopically at 30°C over the indicated times. DIC (differential interference contrast) micrograph images were obtained using an Olympus IX71 microscope with a QIClick digital CCD camera and analyzed using Metamorph software. Strains used were wild type, Δcpc-2 and Δgnb-1. (TIFF) [file pone.0223334.s003.tiff]
