# Supplementary material for: Genetic relationships between the RACK1 homolog cpc-2 and heterotrimeric G protein subunit genes in Neurospora crassa
Source: PLoS One. 2019 Oct 3;14(10):e0223334. doi: 10.1371/journal.pone.0223334 (PMC6776386; doi:10.1371/journal.pone.0223334)
Supplement: S1 Raw Images — All images were captured using a CCD camera. A. Western blot used to generate the top panel of Fig 2A. Cytosolic and particulate fractions were isolated from a protein extract of wild-type strain 74-OR23-1VA as described in the Materials and methods. Samples corresponding to the same volume of original cell extract were subjected to SDS-PAGE and western analysis using plasma membrane ATPase (PMA-1; plasma membrane) antibody. Positions of molecular weight markers are indicated along the right side of the blot. The western blot was treated with chemiluminescence solution and exposed for 5 min. The image was flipped horizontally and darkened for the final figure. The results shown are representative of four biological replicates. B. Western blot used to generate the middle panel of Fig 2A. Cytosolic and particulate fractions were isolated from a protein extract of wild-type strain 74-OR23-1VA as described in the Materials and methods. Samples corresponding to the same volume of original cell extract were subjected to SDS-PAGE and western analysis using arginase (AGA; cytosol) antibody. Positions of molecular weight markers are indicated along the right side of the blot. The western blot was treated with chemiluminescence solution and exposed for 1 min. The image was flipped horizontally and darkened for the final figure. The results shown are representative of four biological replicates. C. Western blot used to generate the bottom panel of Fig 2A. Cytosolic and particulate fractions were isolated from a protein extract of wild-type strain 74-OR23-1VA as described in the Materials and methods. Samples corresponding to the same volume of original cell extract were subjected to SDS-PAGE and western analysis using CPC-2 antibody. Positions of molecular weight markers are indicated along the right side of the blot. The western blot was treated with chemiluminescence solution and exposed for 1 min. The image was flipped horizontally and darkened for the final figure. The res [file pone.0223334.s005.pdf]

A

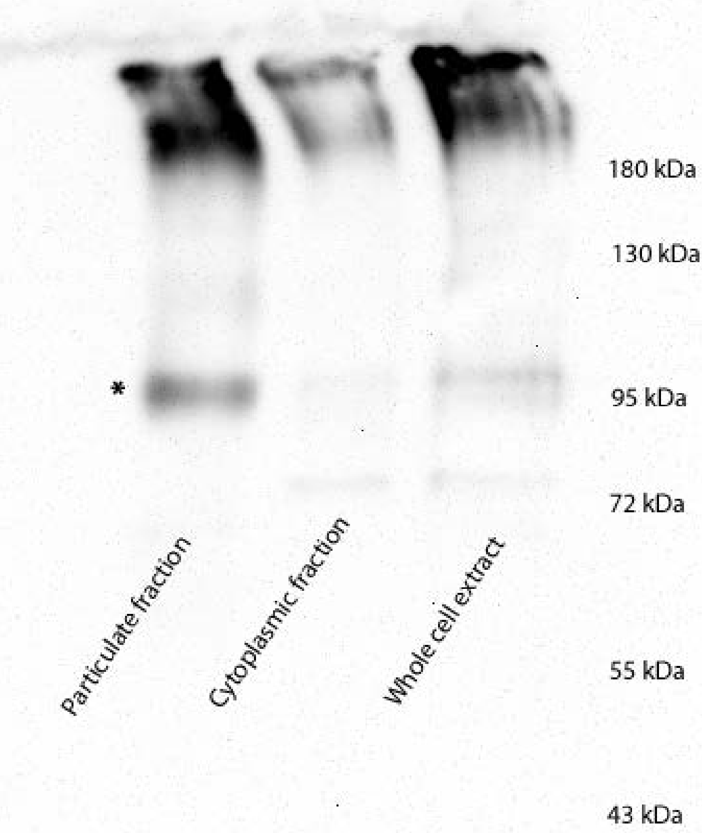

Note:
This image was flipped horizontally in the final figure

Whole blot was darkened in the final figure

Used to generate Fig 2a

Capture method:
Chemiluminescense
5 min exposure

* Denotes PMA-1 band

B

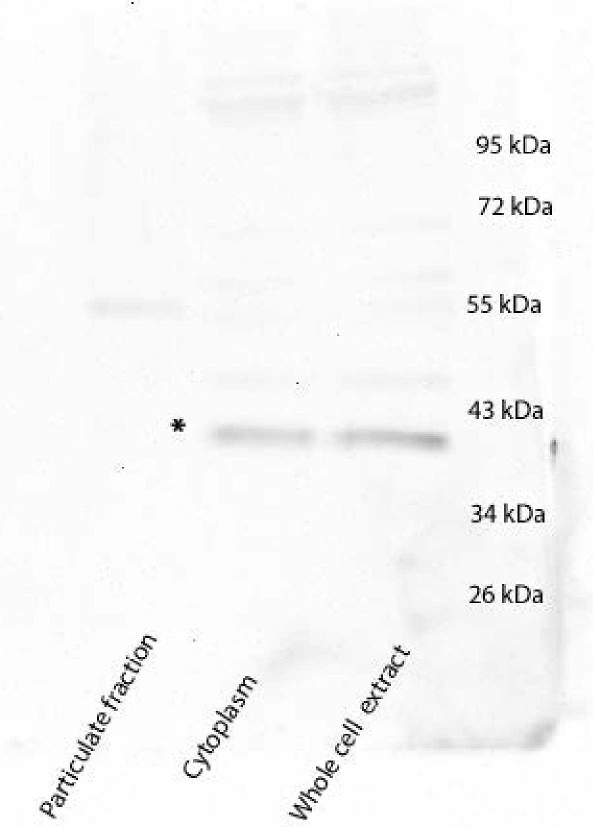

Notes:
Image was flipped horizontally for final figure

Whole blot was darkened for final figure

Used to generate Fig 2a

Method to capture:
chemiluminescence
1 min exposure

* Denotes AGA-1 band

C

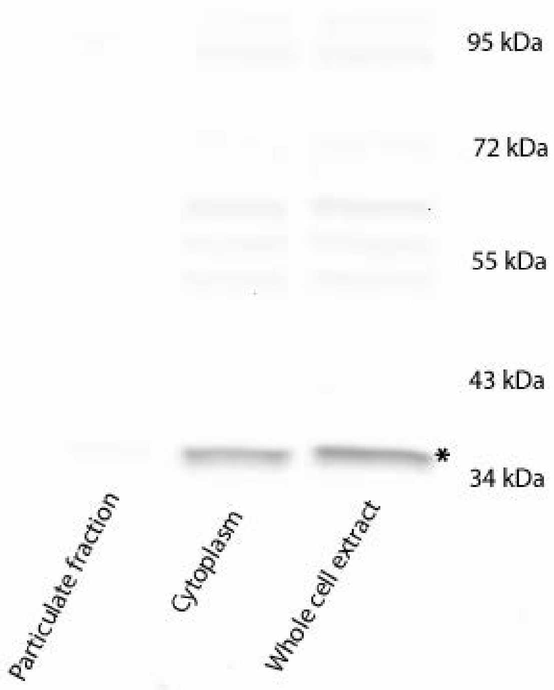

Notes:
Image was flipped horizontally in the final figure

Whole blot was darkened in the final figure

Used to generate Fig 2a

Method to capture:
chemiluminescense
1 min exposure

* Denotes CPC-2 band

D

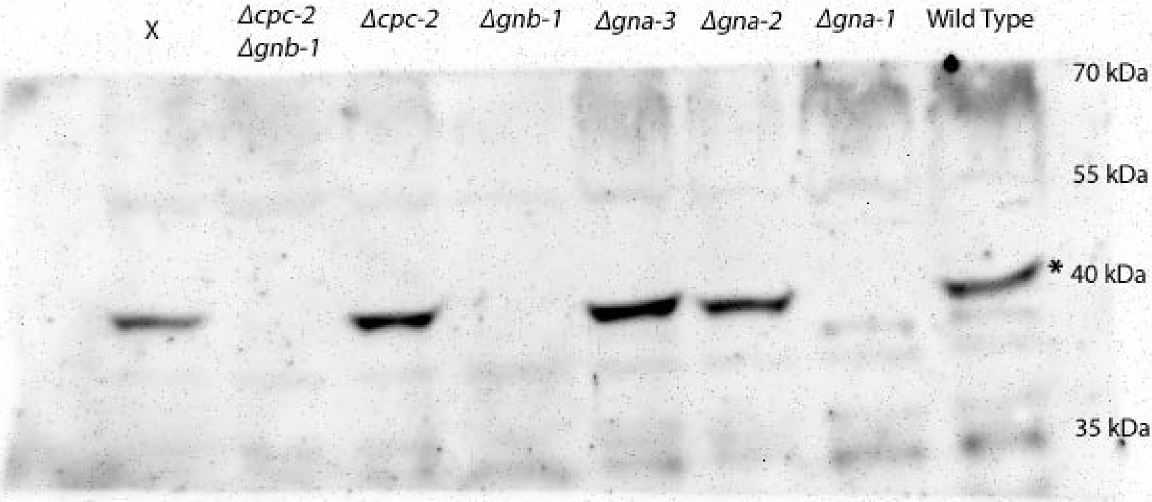

Used to generate Fig 3

Capture Method:
Chemiluminescence
5 minute exposure

* Denotes GNA-1 band

Note: Image was flipped horizontally in final.

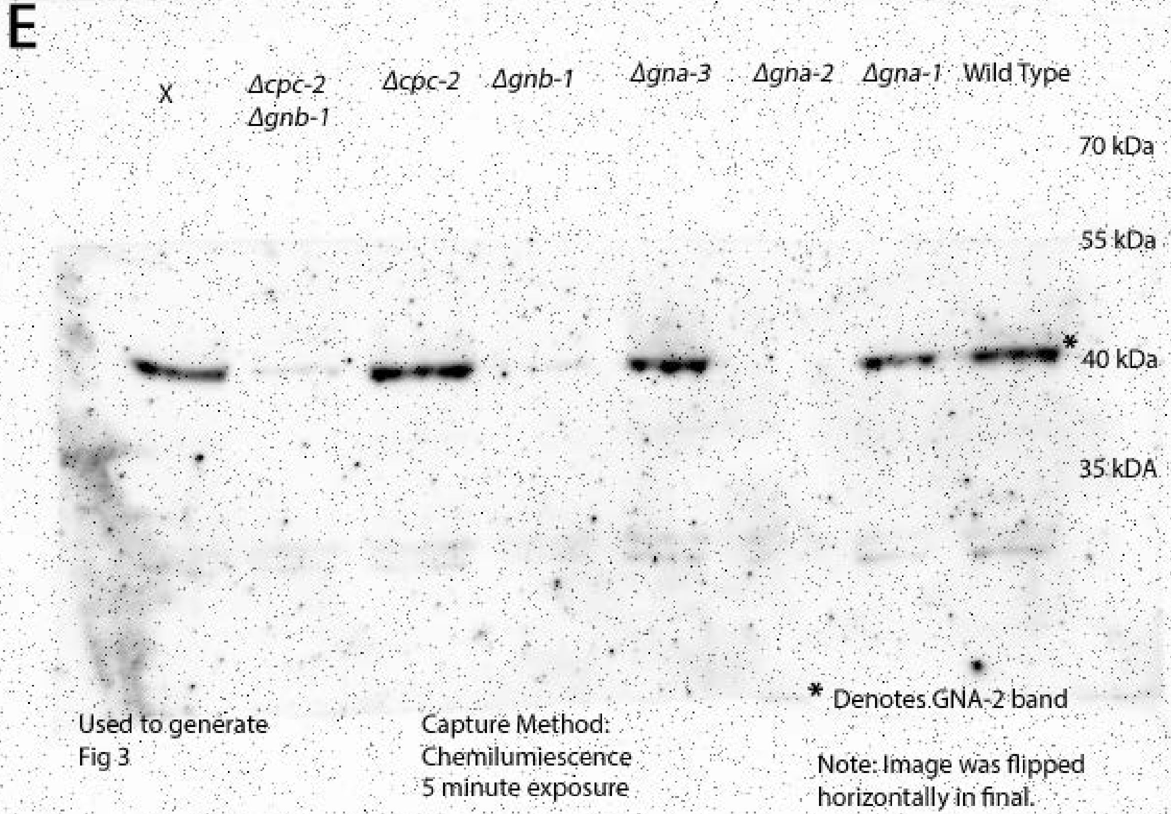
E
X
Δcpc-2
Δgnb-1
Δcpc-2
Δgnb-1
Δgna-3
Δgna-2
Δgna-1
Wild Type
70 kDa
55 kDa
40 kDa
35 kDA
*
* Denotes GNA-2 band
Used to generate
Fig 3
Capture Method:
Chemilumiescence
5 minute exposure
Note: Image was flipped
horizontally in final.

F

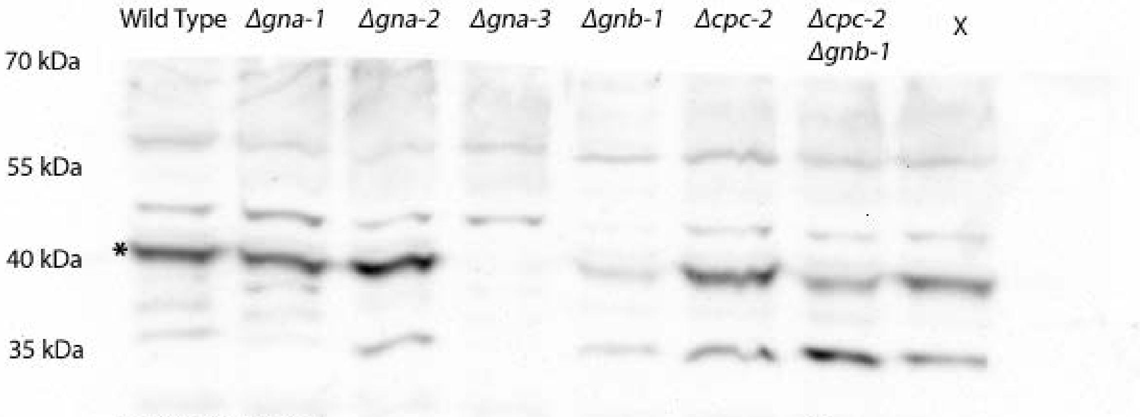

Used to generate Fig 3

Capture Method:
Chemiluminescence
5 minute exposure

* Denotes GNA-3 band

G

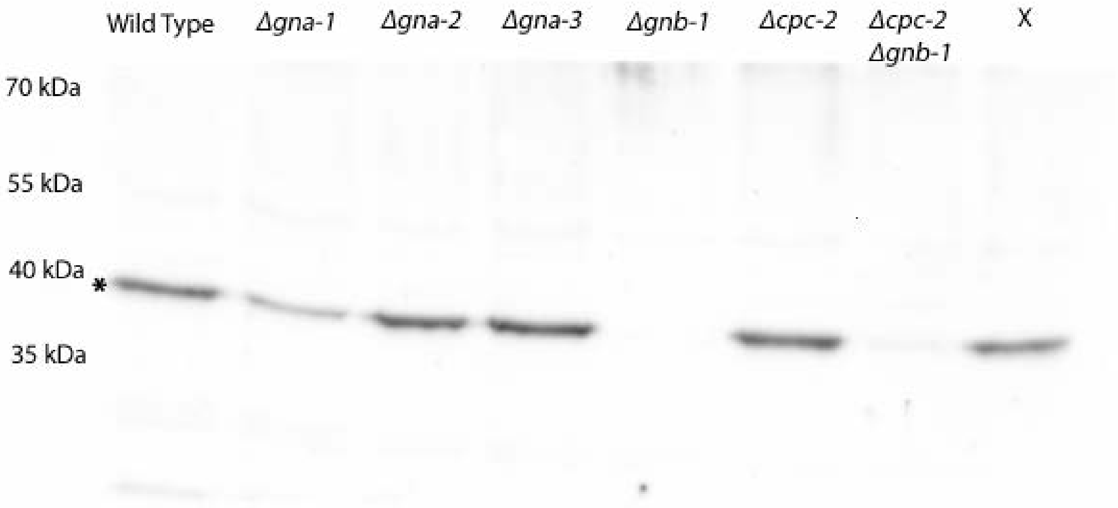

Used to generate
Fig 3

Capture Method:
Chemiluminescence
5 minute exposure

* Denotes GNB-1 band

H

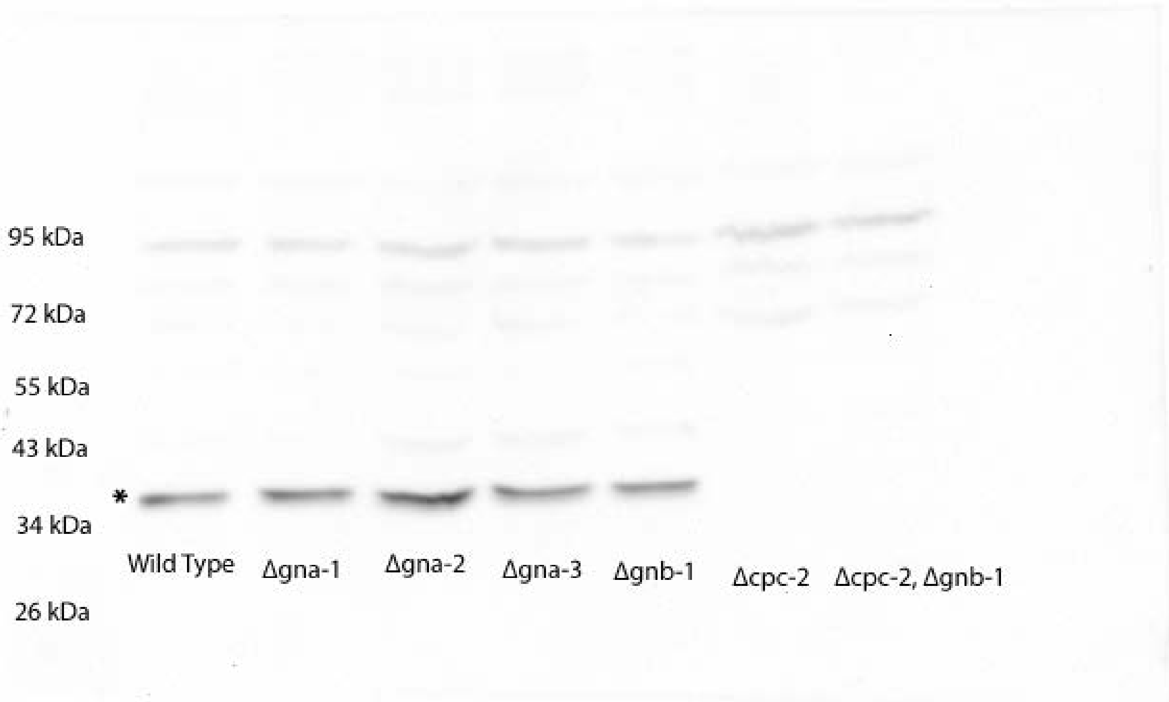

Used to generate
Fig 3

Capture method:
Chemiluminescense
1 min exposure

* Denotes CPC-2 band

Note:
Whole blot was darkened
in the final figure

I

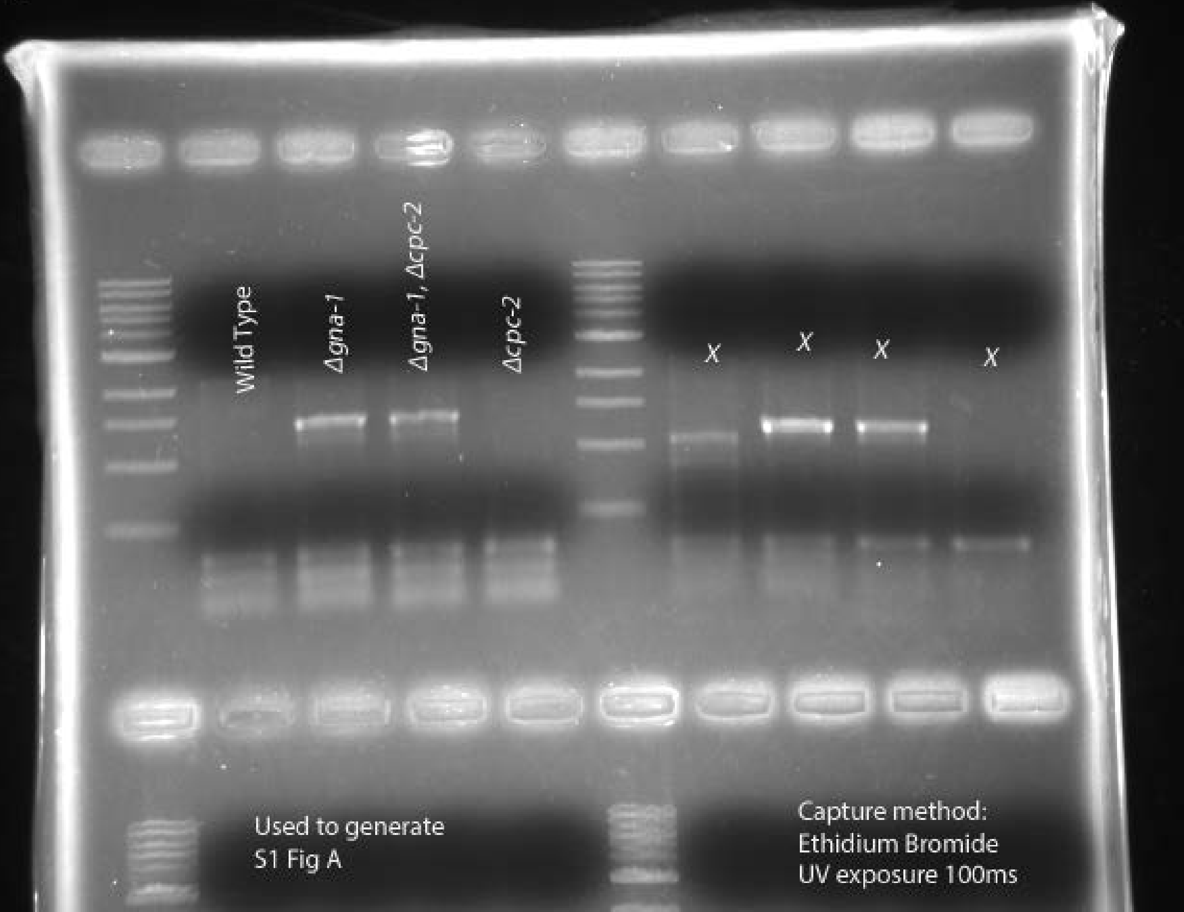
Wild Type
Δgna-1
Δgna-1, Δcpc-2
Δcpc-2
X
X
X
X
Used to generate
S1 Fig A
Capture method:
Ethidium Bromide
UV exposure 100ms

J

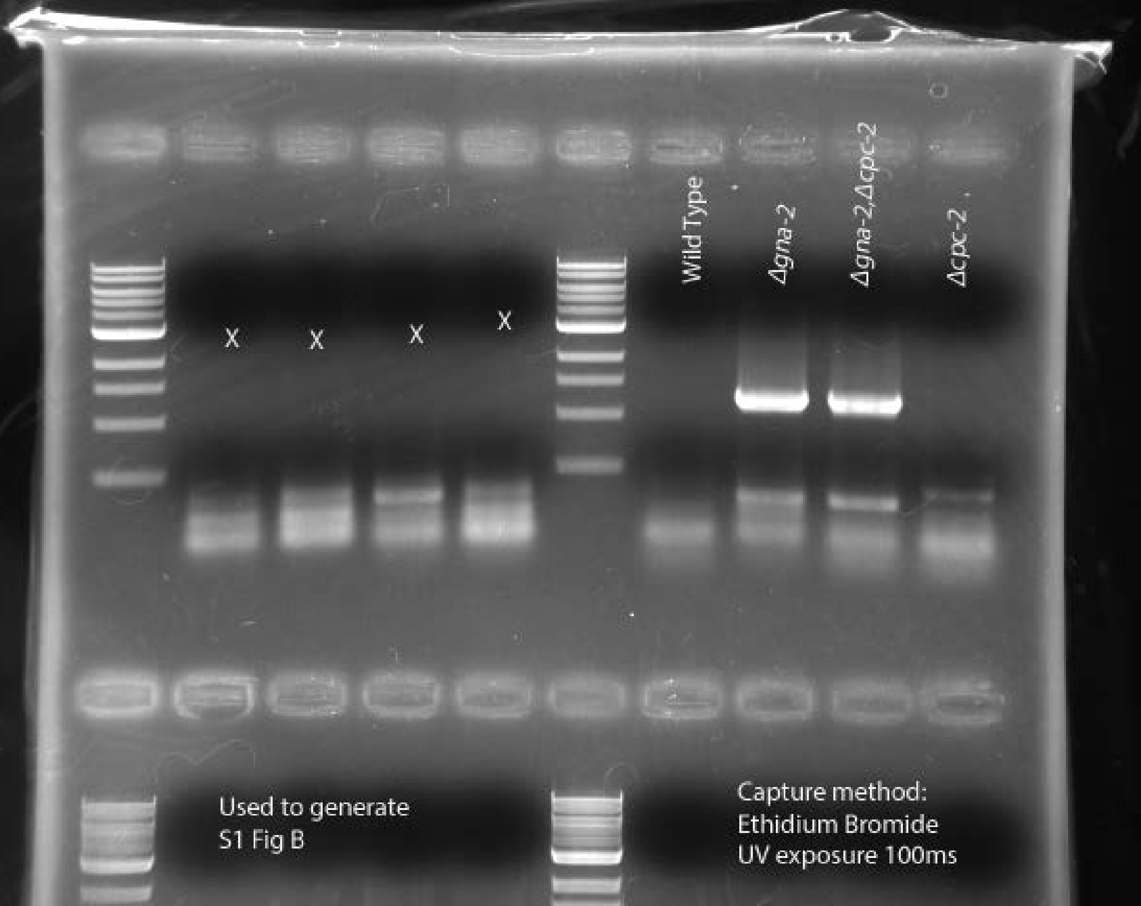
Wild Type
Δgna-2
Δgna-2,Δcpc-2
Δcpc-2
X
X
X
X
Used to generate
S1 Fig B
Capture method:
Ethidium Bromide
UV exposure 100ms

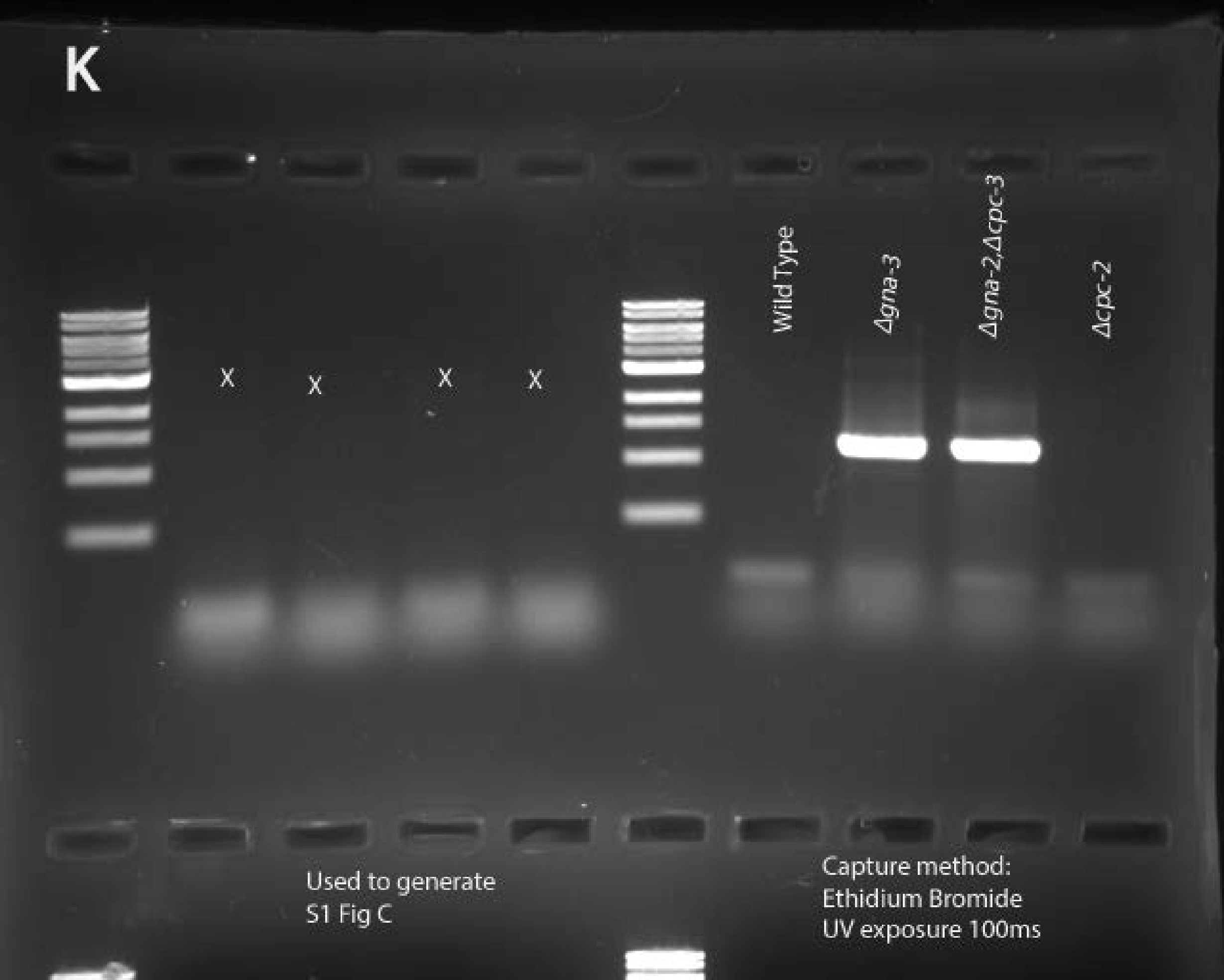
K
X
X
X
X
Wild Type
Δgna-3
Δgna-2,Δcpc-3
Δcpc-2
Used to generate
S1 Fig C
Capture method:
Ethidium Bromide
UV exposure 100ms

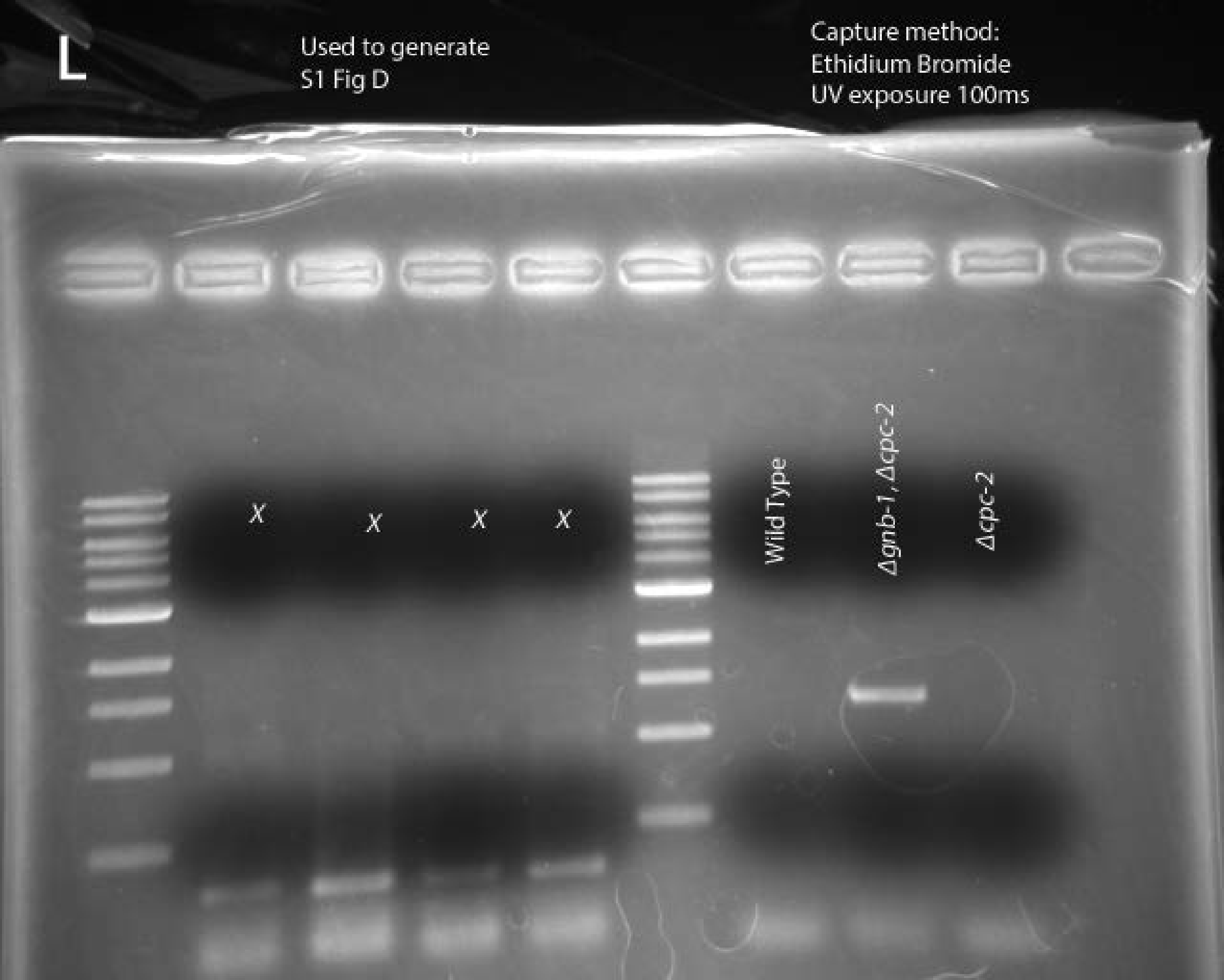
L
Used to generate
S1 Fig D
Capture method:
Ethidium Bromide
UV exposure 100ms
X
X
X
X
Wild Type
Δgnb-1, Δcpc-2
Δcpc-2

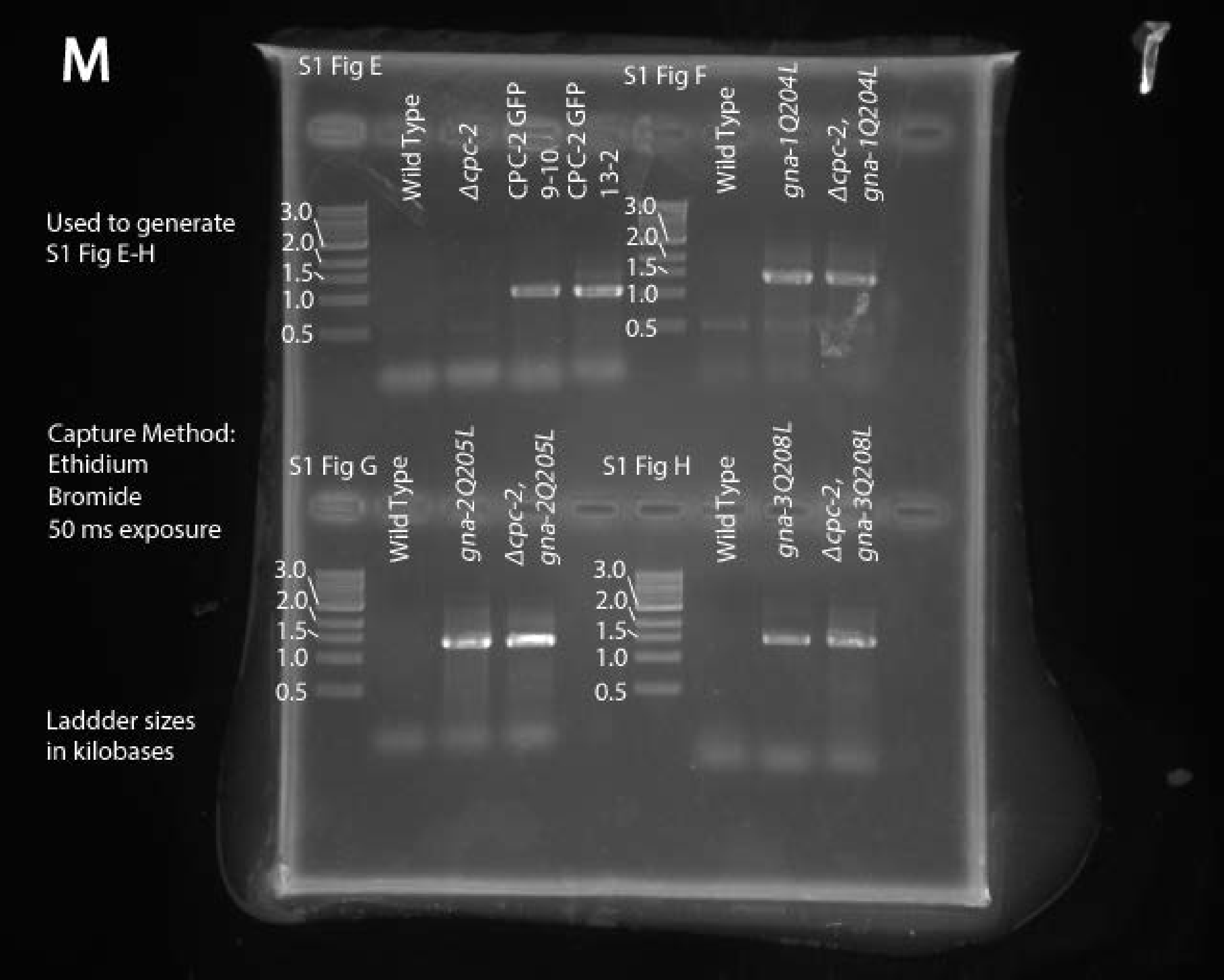
M
Used to generate
S1 Fig E-H
Capture Method:
Ethidium
Bromide
50 ms exposure
Laddder sizes
in kilobases
S1 Fig E
Wild Type
Δcpc-2
CPC-2 GFP
9-10
CPC-2 GFP
13-2
S1 Fig F
Wild Type
gna-1Q204L
Δcpc-2,
gna-1Q204L
S1 Fig G
Wild Type
gna-2Q205L
Δcpc-2,
gna-2Q205L
S1 Fig H
Wild Type
gna-3Q208L
Δcpc-2,
gna-3Q208L
3.0
2.0
1.5
1.0
0.5

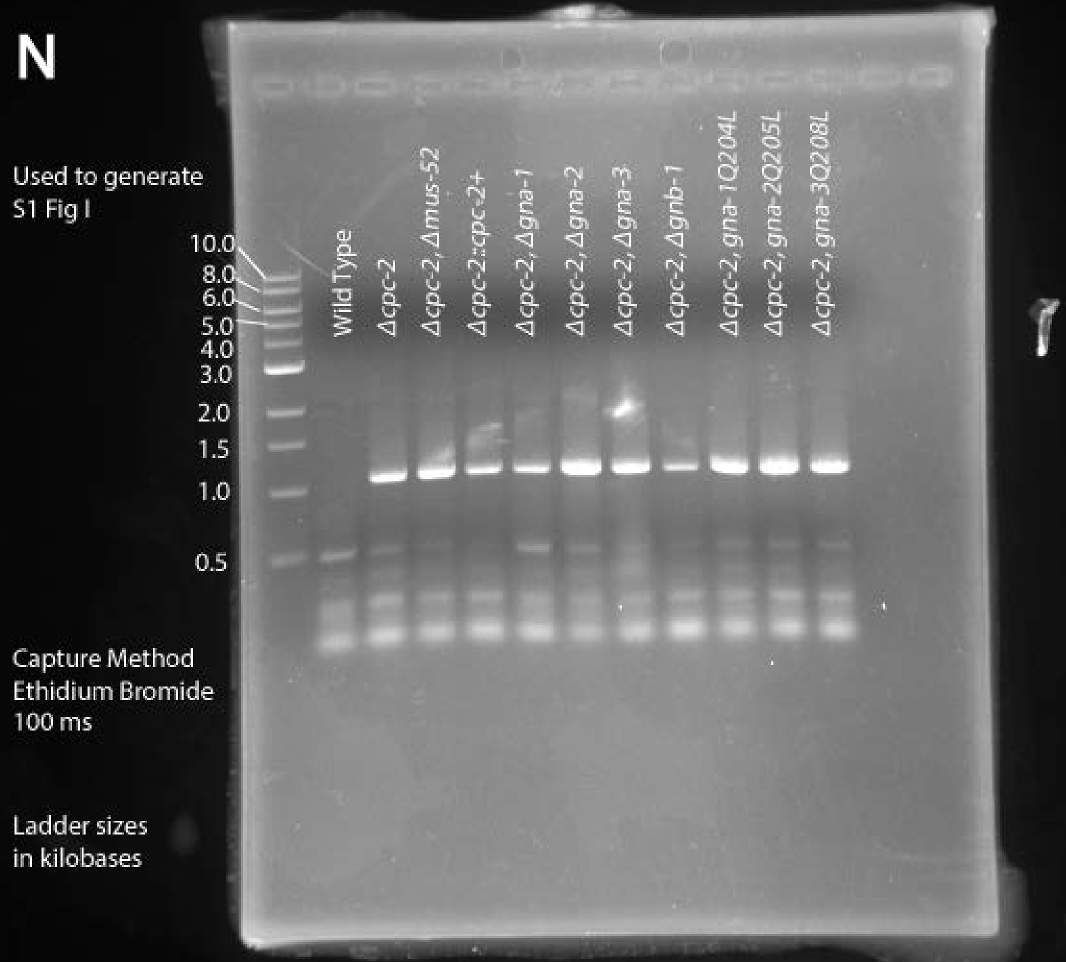
N
Used to generate
S1 Fig I
10.0
8.0
6.0
5.0
4.0
3.0
2.0
1.5
1.0
0.5
Wild Type
Δcpc-2
Δcpc-2, Δmus-52
Δcpc-2::cpc-2+
Δcpc-2, Δgna-1
Δcpc-2, Δgna-2
Δcpc-2, Δgna-3
Δcpc-2, Δgnb-1
Δcpc-2, gna-1Q204L
Δcpc-2, gna-2Q205L
Δcpc-2, gna-3Q208L
Capture Method
Ethidium Bromide
100 ms
Ladder sizes
in kilobases
